# Acute Pain During Intensive Care: Prevalence, Predisposing Factors and Pain Trajectories. A Prospective Multicenter Observational Study

**DOI:** 10.1111/aas.70322

**Published:** 2026-08-24

**Authors:** Aleksandra E. Wlodarczyk‐Abou Elseoud, Maija‐Liisa Kalliomäki, Otto Mäkinen, Annika Laukkanen, Minna Bäcklund, Mitja Lääperi, Katri Hamunen, Anna‐Maria Kuivalainen

**Affiliations:** ^1^ Department of Perioperative and Intensive Care Medicine University of Helsinki and Helsinki University Hospital Helsinki Finland; ^2^ Department of Anesthesiology Tampere University Hospital Tampere Finland; ^3^ Perioperative and Intensive Care Helsinki University Hospital Helsinki Finland; ^4^ Department of Pain Medicine, Neurocenter Helsinki University Hospital Helsinki Finland

**Keywords:** acute pain, analgesics, critical care, pain measurement, pain trajectories

## Abstract

**Background:**

A considerable proportion of patients in the intensive care unit (ICU) experience pain. The objective of this prospective observational cohort study was to investigate the prevalence of pain, factors associated with pain and pain trajectories in ICU patients.

**Methods:**

Critically ill adult patients admitted to four ICUs across two tertiary university hospitals between 2018 and 2020 were recruited. Patients were followed during ICU stay for up to 14 days. Pain was measured using the Numeric Rating Scale (NRS), the Critical Care Pain Observation Tool (CPOT) and the Verbal Rating Scale (VRS). The primary outcome was the prevalence of at least moderate pain at rest defined as NRS ≥ 4, VRS ≥ moderate pain or CPOT ≥ 3. Risk factors for pain were analyzed with mixed effects logistic regression models. To enable a visual analysis of pain prevalence, a trajectory model was constructed based on pain assessment data.

**Results:**

In total, 711 patients participated in the study. Most patients (76.4%) had at least moderate pain at some point during their ICU stay. Female sex, surgical reason for admission and opioids administered during ICU stay were all associated with increased odds for pain, whereas continuous sedation and a higher SAPS II score decreased the odds. The prevalence of at least moderate pain did not decline during ICU stay.

**Conclusion:**

The prevalence of at least moderate pain is relatively high in critically ill patients despite frequent use of opioids in the ICU. Identifying patients at risk for pain may aid in individualising pain management.

**Editorial Comment:**

In this prospective study in a mixed adult ICU cohort, pain prevalence, character and trajectory is described. The majority of cases reported pain at rest, and case factors are presented.

## 1 Introduction

Up to 50% of critically ill patients experience pain during their hospitalisation [1] and in mechanically ventilated patients, the prevalence of pain at rest is even higher [2, 3, 4], despite attempts to standardise the assessment and treatment of pain, agitation and delirium [5].

Inadequately treated pain can have various deleterious effects on critically ill patients [6, 7]. In a retrospective cohort study [8], pain during intensive care unit (ICU) stay was independently associated with increased 30‐day in‐hospital mortality. Furthermore, poorly managed acute pain is associated with the development of persistent pain [9, 10] and increased psychological symptoms [11].

To date, only a few studies have focused on pre‐existing pain, analgesic use and other pain‐specific risk factors prior to ICU admission and their association with acute pain during ICU stay. More data are needed on pain prevalence and pain trajectories in the critically ill. Identifying pain risk factors and describing pain trajectories may enable tailored on‐demand pain management for ICU patients.

The aims of the present study were to examine the prevalence of pain, pain trajectories and risk factors for acute and persistent pain in a cohort of medical and surgical ICU patients. We also describe the current analgesic regimen in the ICU.

## 2 Materials and Methods

### 2.1 Study Design

This prospective observational multicenter cohort study was performed in the mixed ICUs of Helsinki and Tampere University Hospitals. Both are large tertiary teaching hospitals treating adult medical and surgical ICU patients, including those undergoing abdominal, cardiothoracic, vascular, and transplant surgery and trauma cases. The planned enrolment was 1000 patients for sufficient admission subgroup representation.

The ethics committee of Helsinki University Hospital approved the study protocol (Study ID number HUS/2779/2017) on 15 November 2017. Throughout the study, the principles of the Helsinki Declaration were followed. The study was registered at ClinicalTrials.gov (Identifier: NCT03432546; 14 February 2018). Patients or next of kin were approached in person or by telephone to obtain written informed consent at enrolment or within a reasonable time after hospitalisation. Patients permanently unable to consent were excluded.

### 2.2 Patients and Procedures

Patients (≥ 18 years of age) needing ICU care for critical illness or after major elective surgery were eligible. Predefined exclusion criteria were pregnancy, major traumatic brain injury, neurosurgical conditions, estimated survival less than 48 h and major cognitive or psychiatric disorder limiting communication with the patient. Only Finnish or Swedish‐speaking patients were included.

Clinical practice guidelines [5] were followed for the assessment of pain, delirium and sedation. Pain was assessed with the Numeric Rating Scale (NRS, 0–10) or the Verbal Rating Scale (VRS, no pain—unbearable pain). The Critical Care Pain Observational Tool (CPOT, 0–8) [12] was used for patients unable to communicate due to delirium or deep sedation. Pain was assessed at minimum every 2 h using analgesia‐first protocols for pain, sedation and delirium in both centres [5]. The analgesics administered and methods of local or regional analgesia were recorded. Daily opioid doses were converted to oral morphine equivalents (MEQ) (Table S1).

Agitation and sedation were measured with the Richmond Agitation‐Sedation Scale (RASS) [13] and the minimum and maximum scores were recorded daily. Delirium was assessed twice daily using the Intensive Care Delirium Screening Checklist (ICDSC) [14], and considered present if the ICDSC score was 4 or more. Continuous sedation was recorded if administered for 6 h or longer during a calendar day.

Demographics, co‐morbidities, admission diagnosis, Simplified Acute Physiology Score II (SAPS II), mechanical ventilation duration, ICU and hospital length of stay, diagnosis of persistent pain prior to admission, its classification (nociceptive, neuropathic or not known) and pre‐admission analgesic use were collected. Body temperature, C‐reactive protein (CRP), leukocyte counts, vasopressors and inotropes were retrieved from electronic health records. Procedures, complications and invasive devices were recorded daily.

Data were collected for the first 13 calendar days, and additionally on the day of discharge if the ICU stay exceeded 13 days. First ICU day was defined as the period from admission to midnight; subsequent days were counted as calendar days. After pseudonymisation, data were transferred into electronic case report forms (eCRF). Readmitted patients continued in the study. Pain assessments were not collected outside the ICU, consistent with the study's ICU‐focused design. Long‐term follow‐up results will be reported separately upon completion.

### 2.3 Outcomes

The primary outcome was the prevalence of at least moderate pain at rest defined as the highest daily pain value of NRS ≥ 4, VRS ≥ moderate or CPOT ≥ 3 during the stay in the ICU. In addition, we examined factors associated with at least moderate pain and assessed the trajectories of pain, specifically how pain intensity changed during ICU admission. Throughout this paper we refer to at least moderate pain as pain.

### 2.4 Statistics

Demographic data are presented as medians and ranges with counts and percentages.

Since various pain scores are needed in pain assessment due to fluctuations in the patient's consciousness, level of sedation and delirium, pain scores were dichotomised as less than moderate (NRS 0–3, CPOT 0–2 or VRS < moderate pain) and at least moderate (NRS ≥ 4, CPOT ≥ 3 or VRS ≥ moderate pain) to include all available pain data from the three scales. This approach has been published previously [4, 15]. Patients with missing pain assessments were included in the denominator, resulting in a conservative estimate of pain prevalence. Missing data likely reflect gaps in documentation or situations in which pain assessment was not feasible, rather than a true absence of pain. All regression analyses used a complete case approach; missing pain values were never assumed to indicate absence of pain. Pain trajectories were constructed for pre‐specified subgroups defined by sex, age, pre‐ICU opioid use and admission category (surgical vs. medical), which were chosen based on factors known to influence pain.

As the median length of stay in the ICU was 3 days, we modelled the changes in pain for the first 3 days using mixed effects logistic regression with random intercepts for each patient. Covariates for the regression models were pre‐specified based on clinical relevance. *p*‐values were calculated using Satterthwaite's degrees of freedom method. Length of stay filtering was done to prevent potential bias caused by outlier patients having unexpectedly long treatment in the ICU. We fitted the models for the whole observation period and specifically for a priori planned subgroup excluding elective surgical admissions, since a significant fraction of postsurgical elective admissions are relatively short in duration and this population may differ clinically from other critically ill patients. To avoid the over‐influence of outliers, we set the values above the 99th percentile to the 99th percentile (winsorisation) for leukocytes, CRP and MEQ. *p*‐values below 0.05 were considered significant. All the analyses were done with R Statistical Software version 4.3.0. The mixed models were fitted using lme4 R package and the plots were created using ggplot2 R package.

## 3 Results

**FIGURE 1:**
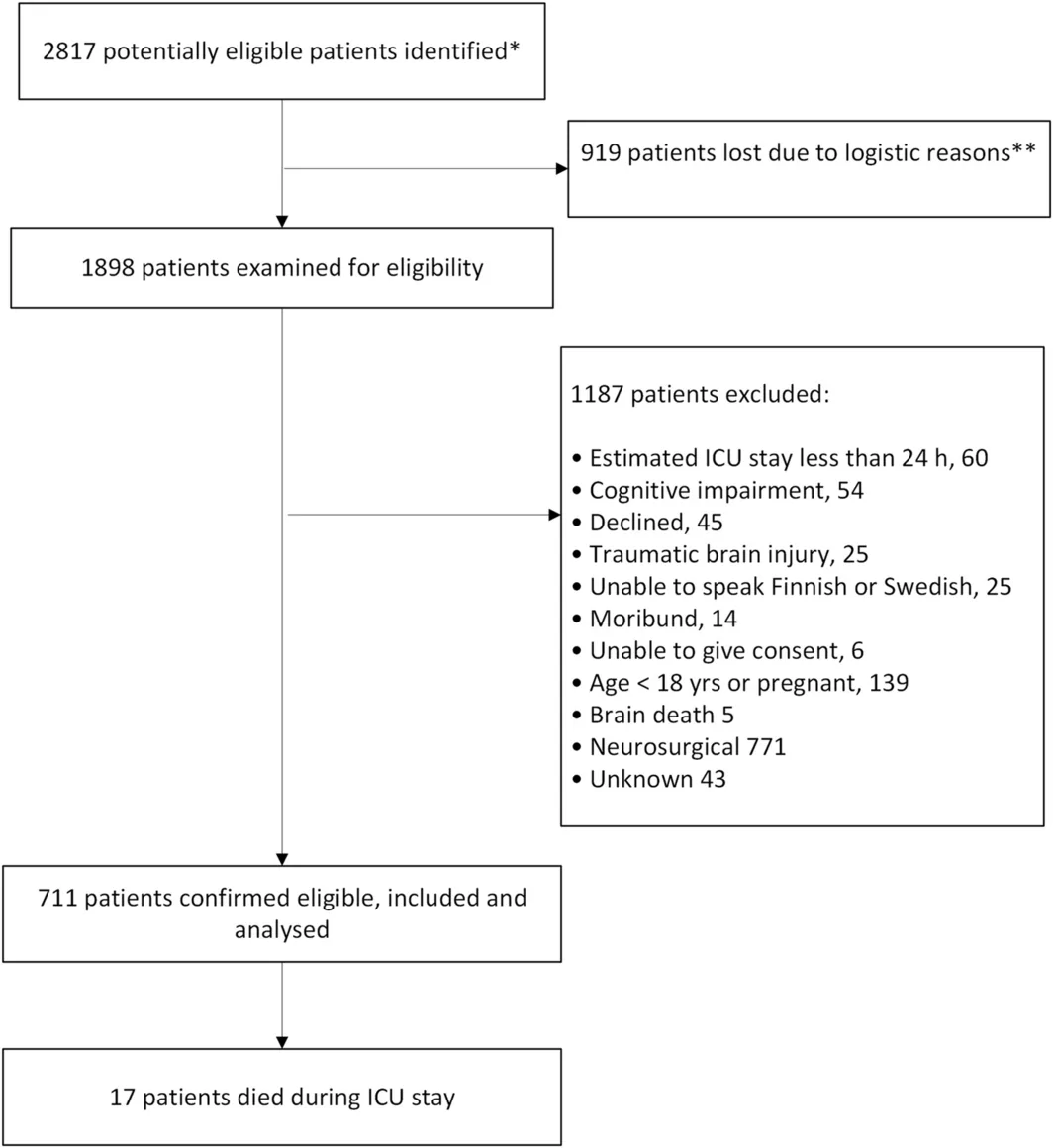
Flow chart of the participants and the various reasons for exclusion. *Potentially eligible patients were defined as all patients admitted to the ICU during the recruitment period. **Patients lost due to logistic reasons were those who were discharged from the ICU before the study team was able to perform eligibility screening.

The recruitment of patients started in December 2018 but was halted in March 2020 due to the COVID 19 pandemic, with a total of 711 patients (Figure 1). The study sample (Tables 1 and S2) was representative of the average adult Finnish ICU population [16], and it was deemed sufficient to form the admission subgroups, enabling statistical analysis to be carried out. Fifteen patients were readmitted to the ICU.

**TABLE 1:** Demographics and characteristics of the study cohort.

Patients, *n*	711
Age, median (IQR; range)	64 (53–71; 18–92)
Female gender, *n* (%)	257 (36.1)
Surgical admission^a^, *n* (%)	494 (69.5)
Main reason for admission to ICU	
Postoperative elective, *n* (%)	273 (38.4)
Postoperative emergency, *n* (%)	127 (17.9)
Severe infection or sepsis, *n* (%)	74 (10.4)
Respiratory distress, *n* (%)	69 (9.7)
Trauma, *n* (%)	37 (5.2)
Cardiac arrest, *n* (%)	26 (3.7)
Pancreatitis, *n* (%)	14 (2.0)
Acute kidney injury, *n* (%)	8 (1.1)
Acute liver failure, *n* (%)	8 (1.1)
Neurologic disorder, *n* (%)	6 (0.8)
Endocrinologic disorder, *n* (%)	10 (1.4)
Heart failure, *n* (%)	7 (1.0)
Other, *n* (%)	52 (7.3)
Comorbidity	
No comorbidity, *n* (%)	276 (38.8)
Diabetes, *n* (%)	164 (23.1)
Cancer, *n* (%)	41 (5.8)
Respiratory disease, *n* (%)	41 (5.8)
Heart failure, *n* (%)	33 (4.6)
Chronic kidney disease *n* (%)	25 (3.5)
Immunosuppression, *n* (%)	64 (9.0)
Liver cirrhosis, *n* (%)	33 (4.6)
Psychiatric disorder, *n* (%)	37 (5.2)
Persistent pain before or at admission^b^, *n* (%)	131 (18.4)
Neuropathic pain^c^, *n* (%)	47 (6.6)
Nociceptive pain^d^, *n* (%)	93 (13.1)
SAPS II score, median (range)	28 (21–38; 0–87)
Length of stay in ICU, days, median (IQR; range)	3 (2–6; 1–121)
Length of mechanical ventilation, hours, median (IQR; range)	5 (0–29; 0–1930)
Length of hospital stay, days, median (IQR; range)	13 (8–22; 2–316)

Abbreviations: ICU, Intensive Care Unit; SAPS II, The Simplified Acute Physiology Score II.

^a^ Admission after surgery, postoperative complications, bleeding, perioperative emergencies, pancreatitis, acute liver failure, trauma.

^b^ Persistent pain (nociceptive, neuropathic or unspecified) persisting or recurring longer than 3 months before ICU admission.

^c^ Persistent pain described as neuropathic (neurological lesion or disease and neuroanatomically plausible pain distribution) diagnosed prior to ICU admission.

^d^ Persistent pain described as nociceptive (pain arising from actual or threatened non‐neural tissue damage) present prior ICU admission.

**FIGURE 2:**
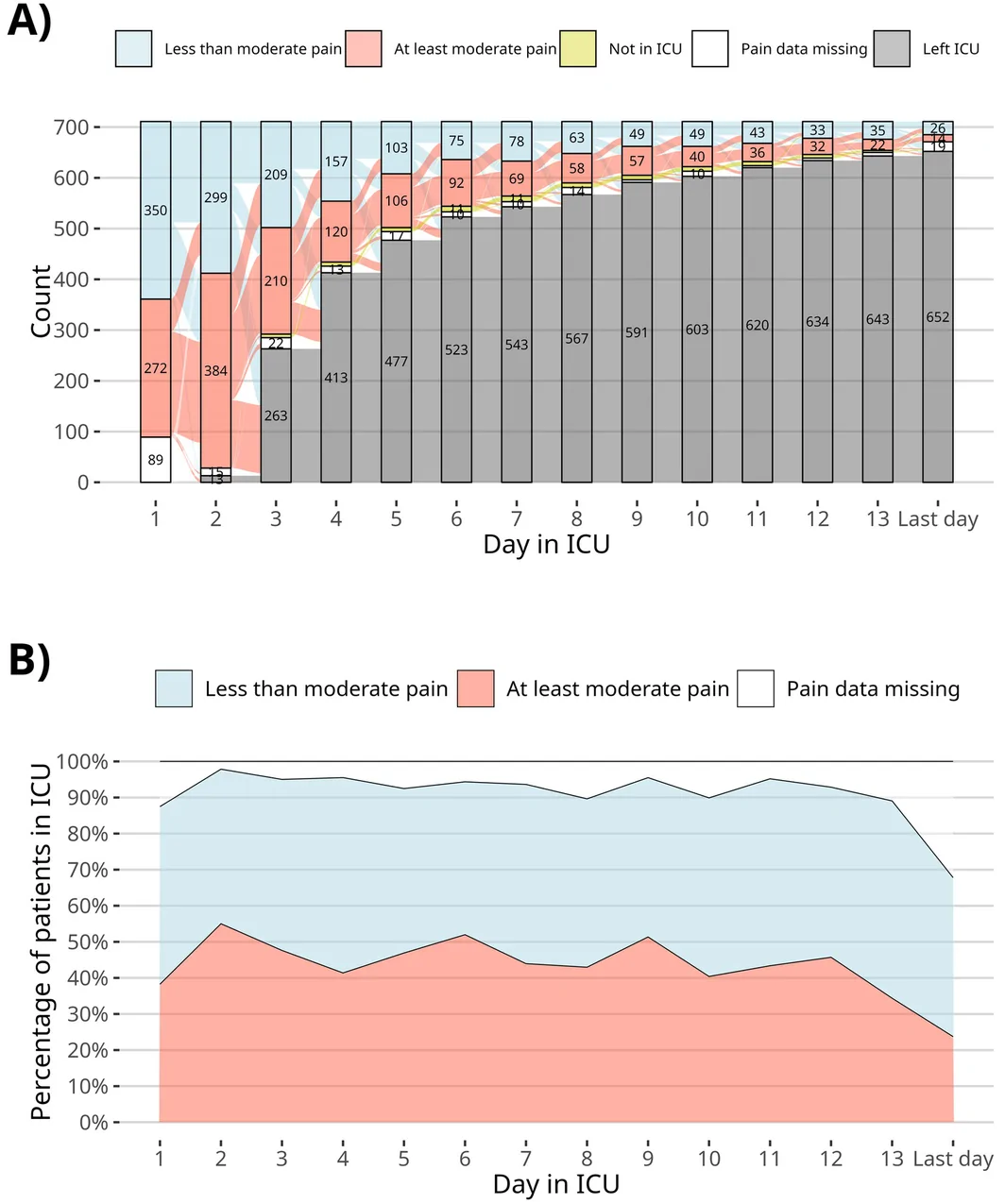
Visualisation of the dichotomised pain variable during the follow‐up period in the ICU. (A) Trajectories of pain in the entire study population. (B) Daily distribution of pain in patients treated in the ICU per evaluation day. Less than moderate pain: NRS < 4, CPOT < 3 or VRS<moderate. At least moderate pain: NRS ≥ 4, CPOT ≥ 3 or VRS ≥ moderate. Not in ICU: Pain data not available due to patient being temporarily treated outside the ICU (e.g., operation room, another unit). Pain data missing: Pain data not available for the evaluation day in the ICU. Left ICU: Pain data no longer available due to discontinuation of ICU treatment. CPOT = Critical Pain Observation Tool, NRS = Numeric Rating Scale, VRS = Verbal Rating Scale.

During the ICU stay, 76.4% (*n* = 543) of the total cohort experienced pain at rest, with rates of 78.7% (*n* = 389) among surgical patients and 71% (*n* = 154) among medical patients. On average, 43.7% of patients in the ICU experienced pain at rest each day, which did not decline during follow‐up (Figure 2). The occurrence of pain was the highest, 55%, on the second day (Figure 2, Table S3) and lowest on the last ICU day, 23.7%.

Opioids and paracetamol were mostly used for pain management (Tables 2 and S4). Median daily total opioid dose varied from 7 to 51 mg MEQ. Except for the first and last ICU day, the opioid dose was over 31 mg MEQ (Table 2). The prevalence of delirium was relatively small, but it increased along with the prolongation of ICU stay (Table S6). Tables S4–S8 present further details regarding critical care.

**TABLE 2:** Features of opioid analgesia during treatment in the ICU.

Day in ICU, *n*	MEQ^a^, median (IQR; range)	MEQ^a^, median, (range)		Strong opioids^b^, *n*		Weak opioids^c^, *n*	
	Whole cohort	Surgical patients	Non‐surgical patients	Surgical	Non‐surgical	Surgical	Non‐surgical
Day 1 (*n* = 711)	21.0 (39; 0–1460)	27 (0–1460)	17.5 (0–186)	382	181	8	2
Day 2 (*n* = 698)	45.0 (64.13; 0–1275)	48 (0–1275)	36.8 (0–385)	389	202	32	9
Day 3 (*n* = 441)	33.0 (60.88; 0–1110)	34.5 (0–1110)	30 (0–332.5)	195	153	13	5
Day 4 (*n* = 290)	39.5 (65.25; 0–885.5)	45 (0–885.5)	33 (0–265.5)	125	115	8	2
Day 5 (*n* = 226)	42.0 (68.69; 0–1960)	48.5 (0–1960)	39 (0–387)	95	90	6	3
Day 6 (*n* = 177)	48.0 (66; 0–369.5)	52.5 (0–302)	44.3 (0–369.5)	73	70	4	1
Day 7 (*n* = 157)	36 (52.13; 0–993.5)	45 (0–993.5)	33 (0–343.4)	65	67	3	1
Day 8 (*n* = 135)	42.0 (60; 0–829)	42.8 (0–829)	40.5 (0–636)	51	58	1	0
Day 9 (*n =* 111)	51.0 (59; 0–1242.5)	55.5 (0–1242.5)	45 (0–266)	42	52	0	1
Day 10 (*n* = 99)	40.5 (47; 0–1508.5)	50.3 (0–1508.5)	37 (0–378)	38	45	2	1
Day 11 (*n* = 83)	37.5 (52; 0–1960)	43.5 (0–1960)	33 (0–750)	31	41	1	0
Day 12 (*n* = 70)	31.5 (52.5; 0–883)	47.5 (0–883)	25.5 (0–474)	25	34	2	0
Day 13 (*n* = 64)	31.5 (43.5; 0–1581)	33 (0–1581)	22.5 (0–297)	25	28	0	1
Last day (*n* = 59)	7.0 (24; 0–912)	6 (0–912)	7.5 (0–154)	15	17	0	0

*Note:* Daily opioid doses in oral morphine equivalents for whole cohort, surgical and non‐surgical subgroups and number of surgical and non‐surgical patients receiving strong and weak opioids on each day of follow up.

Abbreviation: *n* = number of study patients present in the ICU on each day of follow up.

^a^ Daily cumulative opioid dose defined as the median and range (min, max) of total sum of all opioids converted to oral morphine equivalents (MEQ) in milligrammes (mg).

^b^ Strong opioids: fentanyl, oxycodone, alfentanil, remifentanil, sufentanil, methadone.

^c^ Weak opioids: tramadol, codeine; moderate opioid: buprenorphine.

Both unadjusted and adjusted models for the first 3 days of follow‐up showed female gender, surgical reason for admission and opioid dose during ICU treatment as factors associated with pain at rest in the ICU (Table 3). A pre‐admission history of psychiatric disorder and chronic neuropathic pain were only associated with pain at rest in the unadjusted model. However, continuous sedation and higher disease severity in SAPS II decreased odds for pain in both the unadjusted and adjusted models. Higher age and noradrenaline dose over 0.1 μg/kg/min decreased odds for pain in the unadjusted model, but the effect of age was lost in the adjusted models and the noradrenaline dose was dropped from the adjusted models due to overfitting. Missingness in regression covariates was minimal (Table S10).

**TABLE 3:** Mixed‐effects logistic regression results for pain during the first three follow‐up days.

Variable	Unadjusted model		Adjusted model	
	OR (95% CI)	*p*	OR (95% CI)	*p*
Age (logarithmised)	0.52 (0.30–0.89)	0.017	1.53 (0.78–2.98)	0.22
Female gender	1.51 (1.09–2.08)	0.012	1.61 (1.13–2.30)	< 0.01
BMI^a^	1.00 (0.97–1.02)	0.82	NC	NC
No comorbidities^b^	0.96 (0.70–1.32)	0.81	NC	NC
Diabetes^c^	1.12 (0.78–1.62)	0.53	1.34 (0.91–1.98)	0.14
Cancer^d^	0.93 (0.65–1.31)	0.67	NC	NC
Psychiatric disorder^e^	2.54 (1.24–5.22)	0.011	1.72 (0.76–3.90)	0.19
Persistent pain^f^	1.52 (1.02–2.26)	0.039	NC	NC
Nociceptive pain^g^	1.23 (0.78–1.93)	0.38	0.80 (0.48–1.34)	0.40
Neuropathic pain^h^	2.32 (1.23–4.36)	< 0.01	1.49 (0.71–3.15)	0.29
Opioid use^i^	0.87 (0.43–1.73)	0.68	1.03 (0.33–3.27)	0.95
Surgical ICU admission^j^	2.92 (2.10–4.06)	< 0.01	2.42 (1.61–3.65)	< 0.01
SAPS II, per 10 units^k^	0.76 (0.68–0.85)	< 0.01	0.86 (0.74–0.99)	0.042
Vasopressor^l^	0.73 (0.54–0.99)	0.04	NC	NC
Sedation^m^	0.74 (0.55–0.98)	0.034	0.62 (0.43–0.90)	0.011
Dexmedetomidine	1.42 (0.86–2.34)	0.17	1.93 (1.08–3.46)	0.027
MEQ, per 10 units^n^	1.17 (1.13–1.21)	< 0.01	1.22 (1.17–1.27)	< 0.01
Regional analgesia^o^	0.96 (0.72–1.28)	0.79	1.22 (0.87–1.71)	0.26
Delirium^p^	1.23 (0.73–2.09)	0.44	NC	NC
Fever^q^	1.25 (0.92–1.68)	0.15	NC	NC
Leukocytosis, per 10 units^r^	0.98 (0.76–1.26)	0.86	1.18 (0.91–1.54)	0.21
CRP, per 10 units^s^	1.00 (0.99–1.01)	0.92	1.00 (0.99–1.01)	0.96

*Note:* Number of observations included in the adjusted model was 1.582.

Abbreviation: NC, not calculated.

^a^ Body mass index in kg/m^2^.

^b^ No diagnosed diseases prior ICU admission.

^c^ Diabetes mellitus diagnosed prior to ICU admission.

^d^ Malignant tumour or leukaemia diagnosed prior to ICU admission.

^e^ Psychiatric disease defined as fulfilling ICD10 diagnostic criteria prior to ICU admission.

^f^ Persistent pain (nociceptive, neuropathic or unspecified) diagnosis prior to ICU admission.

^g^ Persistent pain described as nociceptive (pain arising from actual or threatened non‐neural tissue damage) is present prior to ICU admission.

^h^ Persistent pain described as neuropathic (neurological lesion or disease and neuroanatomically plausible pain distribution) diagnosed prior to ICU admission.

^i^ Regular use of prescription opioids prior to ICU admission.

^j^ Admission after surgery, postoperative complications, bleeding, perioperative emergencies, pancreatitis, acute liver failure, trauma.

^k^ Simplified Acute Physiology Score II.

^l^ Noradrenalin administered > 0.1 μg/kg/min over 1 h per calendar day.

^m^ Continuous administration of a sedative agent (propofol, benzodiazepines, *α* ^2^‐agonists, barbiturates, S‐ketamine or inhalation agent) for over 6 h in calendar day in ICU (excluding sedatives administered outside of ICU).

^n^ Opioid dose in MEQ (oral morphine equivalents) administered in ICU per calendar day.

^o^ Local, regional or neuraxial anaesthesia administered in ICU per calendar day.

^p^ Intensive Care Delirium Screening Checklist ≥ 4 points during calendar day in ICU.

^q^ Body temperature exceeding 38°C at any time during calendar day.

^r^ Leukocyte count > 8.2^9^/L during calendar day in ICU.

^s^ CRP (C Reactive protein) > 100 mg/L during calendar day ICU.

Results for the whole period of follow‐up showed similar findings with three exceptions. Fever and the use of regional analgesia were predictors of pain only in the unadjusted model, whereas continuous sedation was associated with a decrease in the occurrence of pain only in the multi‐variable model. In a model excluding elective surgical patients, the results did not differ from the whole study cohort analysis except for female gender, which was associated with pain only in the adjusted model of the full follow‐up (Table S9A–C). Pain trajectories in the subgroup of patients, categorised by the risk factors for developing pain, are presented in Figures S1–S8.

## 4 Discussion

In our cohort, prevalence of pain at rest was 76.4%, which is higher than in previously reported studies [4, 8, 17]. The prevalence of pain at rest varied daily but exceeded 40% on most follow‐up days, in contrast to a previous study [15], where it decreased over 6‐day follow‐up. Female sex, surgical reason for admission and opioid dose were associated with more pain at rest in the adjusted model.

In the overall cohort, pain prevalence was highest on the second follow‐up day. Similarly, among surgical patients, the highest prevalence of pain was observed on the second ICU day. Since 63.5% of surgical patients underwent major procedures on the ICU admission day, the second ICU day represented the first postoperative day when pain is typically most frequent [18]. Furthermore, the majority of intubated and sedated surgical patients admitted late in the evening had their initial pain score documented on the second calendar day, potentially affecting the results.

Subgroup analysis demonstrated differing pain trajectories across patient groups (Figures S1–S8). In females and in patients with regular opioid use prior to ICU admission, pain prevalence peaked on the second follow‐up day as well as on Days 11 and 12 of follow‐up. This is in line with a previous finding, where patients with prior opioid use have been shown to require higher opioid doses for effective analgesia, and their pain management may take up to three times longer compared with opioid‐naïve patients [19]. Vasilopoulos et al. [20] demonstrated that postoperative pain in women follows a trajectory of sustained high intensity over the first postoperative week, which, together with the potential development of opioid‐induced hyperalgesia during the ICU stay [21], may contribute to the secondary pain peak in the second week of ICU admission. These findings highlight the importance of adjusting pain management throughout the ICU stay.

Among surgical ICU patients, the prevalence of any pain has been reported to be 77%, and at least moderate pain was present in at least 50% [22]. In our study, the prevalence of pain in the surgical group was higher, 78.7%. For medical ICU patients, previous studies have reported a pain prevalence of approximately 50% [17], whereas in our study, it was 71%, indicating a surprisingly high burden of pain in this group. Not all studies have observed a significant difference in pain prevalence between surgical and medical ICU patients [7, 17]. High prevalence of pain observed among medical patients in our study may result from a long observation period and frequent pain assessments, both of which enhance detection sensitivity [23], as well as by the less frequent use of preventive analgesia in this patient group. More information is needed to determine the reasons for pain in medical ICU patients to improve their pain management.

Tissue trauma and related inflammation are one of the leading causes of pain in surgical patients. Inflammation, a factor known to facilitate nociception [24], also occurs in non‐surgical conditions. However, in our cohort, fever was the only inflammatory marker associated with pain and only in the unadjusted whole‐stay model. Leukocytes or CRP were not statistically significant, though inflammation contributes to pain sensitisation [24].

In our study, female gender was associated with pain at rest, as in Ayasrah et al. [2], although contradicting findings exist [1, 8, 15]. Several studies examining post‐surgical pain [25] have shown that women are at a higher risk and exhibit greater sensitivity to pain. The reasons for this could be female hormonal features influencing nociception, psychosocial factors and the divergence in response to pharmacological pain management between genders, among others [26]. Other patient‐related risk factors shown to increase pain during ICU stay are young age [1, 27], which increased the odds for pain in our unadjusted model.

Higher SAPS II score and vasopressor dose were associated with less pain at rest, suggesting severely ill patients may report less pain, possibly due to fewer or delayed pain assessments until stabilisation. Similar [15] and contradicting findings have been reported [8]. In addition, most severely ill patients might express pain differently than other ICU patients, resulting in lower pain scores.

In our cohort, the use of sedation independently decreased the odds for pain. Deep sedation, especially with propofol or midazolam, makes pain assessment more difficult [28]. Sedated patients often appear to be in less pain compared to those not receiving sedatives [15]. Sedatives are known to affect experimental pain levels [29], with dexmedetomidine showing an opioid sparing effect [30] and ketamine providing analgesic benefits [31].

Our results indicate considerable use of opioids, with over 80% of patients receiving opioids on most days, as reported by others [32]. Opioid dose was associated with pain at rest, reflecting that opioids are readily used in patients who specifically report pain. Opioids, especially in high doses and administered long‐term, may lead to the development of hyperalgesia, tolerance [24] and withdrawal syndromes [33], further aggravating pain. In this study, it was not possible to determine the cause‐effect relationship between observed opioid dosage and experienced pain, but Olsen et al. [15] have observed a similar relationship.

Although opioid‐based sedation seems to be more beneficial than sedation alone [34], our results indicate that the effectiveness of opioids as the primary pain treatment remains uncertain. After intensive care, patients often continue using opioids at higher doses than before ICU admission, which is associated with elevated mortality rates [35]. The impact of opioid use during ICU stay on the development of persistent pain after recovery from critical illness remains unclear [10]. Alongside other significant outcomes, such as mortality, this highlights the need for further research. Consequently, opioids should be administered cautiously in critically ill patients.

Multimodal approach is recommended to optimise pain control while minimising risks [36]. However, critical illness can alter drug pharmacokinetics [37], and introduce contraindications limiting the use of neuraxial analgesia [38] or use of non‐opioid pain medications [39]. These constraints were reflected in our study, where 22.8% of patients received epidural analgesia, and regional analgesia techniques were rarely used. The use of NSAIDs and other non‐opioid medication for pain control remained low (Table S5B). New interventional studies are needed to identify patients who benefit most from opioid‐sparing techniques and those for whom opioids are least harmful.

The signs of pain, agitation and delirium in the ICU are often medicated despite incomplete assessment [32]. The systematic evaluation and management of pain and sedation decrease pain intensity [40] and incidence [4, 7], duration of mechanical ventilation [4, 15] and ICU stay [15], agitation and nosocomial infections [4] and serious haemodynamic and ventilatory adverse events [7]. A higher nurse‐to‐patient ratio further facilitates better pain management [41], highlighting the importance of both protocol and organisational factors in optimising analgesia in critically ill patients.

The strengths of this study are the long period of observation during ICU stay and the significant number of mixed ICU patients. The study employed wide inclusion criteria, enabling recruitment of various patient groups and minimising selection bias. The follow‐up period for assessing pain prevalence significantly exceeded the median duration of intensive care treatment, allowing us to effectively capture pain occurrences.

Our study has several limitations. The pre‐specified sample size was not achieved due to early termination of inclusion because of the COVID‐19 pandemic. Excluding patients with neurosurgical conditions might have influenced results in the surgical cohort. The median SAPS score of 28 and relatively low mortality may reflect the exclusion of patients not expected to survive 48 h, and results may therefore not be generalisable to sicker ICU populations. Data from outside the ICU was not collected for readmitted patients, and only sedation lasting over six continuous hours in a single calendar day was considered. The number of pain assessments varied between patients due to differences in admission time and incomplete documentation, which may have introduced bias in pain prevalence estimates. Dichotomisation of pain scores was necessary to pool data from three different pain scales but may have influenced the precision of our results.

## 5 Conclusion

Pain is a significant problem affecting patients during their treatment in the ICU. High‐risk patients, including women, surgical patients and those with persistent pain, should be identified for better pain monitoring and individualised analgesic strategies, recognising that pain management may require adjustment throughout the ICU stay.

Future studies should explore the long‐term impact of pain in ICU on the development of persistent ICU‐related pain and the quality of life after critical illness. In addition, long‐term effects of opioid administration and patterns of opioid consumption in ICU need to be further evaluated.

## Author Contributions

**Anna‐Maria Kuivalainen**, **Katri Hamunen** and **Maija‐Liisa Kalliomäki:** study design. **Anna‐Maria Kuivalainen**, **Aleksandra E. Wlodarczyk‐Abou Elseoud** and **Maija‐Liisa Kalliomäki:** patients' recruitment and data collection. **Aleksandra E. Wlodarczyk‐Abou Elseoud**, **Anna‐Maria Kuivalainen**, **Katri Hamunen** and **Annika Laukkanen:** data screening. **Aleksandra E. Wlodarczyk‐Abou Elseoud**, **Anna‐Maria Kuivalainen** and **Mitja Lääperi:** statistical analysis. All authors participated in abstract and manuscript screening as well as assessing risk of bias. The definitive manuscript was approved by all authors.

## Funding

Aleksandra E. Wlodarczyk‐Abou Elseoud received funding for the study from The Finnish Association for the Study of Pain and Anna‐Maria Kuivalainen was granted funding from The Finnish Society of Intensive Care and The Paulo Foundation. For the remaining authors, no funding was granted.

## Ethics Statement

The ethics committee of Helsinki University Hospital approved the study protocol (Study ID number HUS/2779/2017) on 15 November 2017.

## Consent

Written informed consent was obtained from each patient or, when not feasible, from next of kin, with deferred consent procedures approved by the institutional ethics committee.

## Conflicts of Interest

The authors declare no conflicts of interest.

## Supporting information


**Table S1:** Opioid conversion ratios.
**Table S2:** Pain medication used for chronic pain in the study cohort before ICU admission.
**Table S3:** Percentage values of the daily distribution of pain in ICU patients by evaluation day.
**Table S4:** Daily opioid doses converted to oral morphine equivalents.
**Table S5:** (A) Features of non‐opioid pain alleviation during treatment in the ICU. (B) Number of ICU patients receiving non‐opioid medication categorised by surgical and non‐surgical patient groups.
**Table S6:** Use of continuous sedation and neuromuscular blocking agents, level of sedation and prevalence of delirium during treatment in the ICU.
**Table S7:** Medical devices patients had on each day of follow‐up in the ICU.
**Table S8:** Procedures performed during follow‐up in the ICU and number of complications.
**Table S9:** (A) Results of the mixed effects logistic regression models of pain at rest during the whole period of follow‐up in the ICU. Variables were selected for the adjusted model based on clinical relevance and the results of the univariable model. (B) Results of the mixed effects logistic regression models of pain at rest for the first 3 days of follow‐up in the ICU, excluding elective postoperative patients. Variables were selected for the adjusted model based on clinical relevance and the results of the univariable model. (C) Results of the mixed effects logistic regression models of pain at rest during the whole period of follow‐up in the ICU, excluding elective postoperative patients. Variables were selected for the adjusted model based on clinical relevance and the results of the univariable model.
**Table S10:** Missingness of the variables included in the 3‐day follow up regression model for the whole cohort when the combined pain outcome is available.
**Figure S1:** Visualisation of the dichotomized pain at rest variable during the follow up period in the ICU for the subgroup of surgical patients. (A) Trajectories of pain shown as patient counts. (B) Daily distribution of pain in surgical patient subgroup treated in ICU per evaluation day.
**Figure S2:** Visualisation of the dichotomized pain at rest variable during the follow up period in the ICU for the subgroup of non‐surgical patients. (A) Trajectories of pain shown as patient counts. (B) Daily distribution of pain in non‐surgical patient subgroup treated in ICU per evaluation day.
**Figure S3:** Visualisation of the dichotomized pain at rest variable during the follow up period in the ICU for the subgroup of patients aged 64 years or older. (A) Trajectories of pain shown as patient counts. (B) Daily distribution of pain in subgroup of patients ≥ 64 years old treated in ICU per evaluation day.
**Figure S4:** Visualisation of the dichotomized pain at rest variable during the follow‐up period in the ICU for subgroup of patients aged under 64 years. (A) Trajectories of pain shown as patient counts. (B) Daily distribution of pain in subgroup of patients aged < 64 years treated in the ICU per evaluation day.
**Figure S5:** Visualisation of the dichotomized pain at rest variable during follow‐up period in the ICU for subgroup of male patients. (A) Trajectories of pain shown as patient counts. (B) Daily distribution of pain in subgroup of male patients treated in the ICU per evaluation day.
**Figure S6:** Visualisation of the dichotomized pain at rest variable during follow‐up period in the ICU for subgroup of female patients. (A) Trajectories of pain shown as patient counts. (B) Daily distribution of pain in subgroup of female patients treated in the ICU per evaluation day.
**Figure S7:** Visualisation of the dichotomized pain at rest variable during follow‐up period in the ICU for subgroup of opioid‐naive patients (no prior opioid use before ICU admission). (A) Trajectories of pain shown as patient counts. (B) Daily distribution of pain in subgroup of opioid‐naive patients treated in the ICU per evaluation day.
**Figure S8:** Visualisation of the dichotomized pain at rest variable during follow‐up period in the ICU for subgroup of patients regularly using opioids before ICU admission. (A) Trajectories of pain shown as patient counts. (B) Daily distribution of pain in subgroup of patients regularly using opioids before ICU admission treated in the ICU per evaluation day.

## Data Availability

The datasets generated and/or analysed during the current study are not publicly available due to limitations in participant consent and the absence of approval for dataset publication from the institutional ethics committee.

## References

[1] N. A. Desbiens , A. W. Wu , S. K. Broste , et al., “Pain and Satisfaction With Pain Control in Seriously Ill Hospitalized Adults: Findings From the SUPPORT Research Investigations. For the SUPPORT Investigators. Study to Understand Prognoses and Preferences for Outcomes and Risks of Treatmentm,” Critical Care Medicine 24, no. 12 (1996): 1953–1961. 8968261 10.1097/00003246-199612000-00005

[2] S. M. Ayasrah , “Pain Among Non‐Verbal Critically Ill Mechanically Ventilated Patients: Prevalence, Correlates and Predictors,” Journal of Critical Care 49 (2019): 14–20. 30339991 10.1016/j.jcrc.2018.10.002

[3] R. M. Fink , M. B. Makic , A. W. Poteet , M. B. F. Makic , and K. S. Oman , “The Ventilated Patient's Experience,” Dimensions of Critical Care Nursing 34, no. 5 (2015): 301–308. 26244246 10.1097/DCC.0000000000000128

[4] G. Chanques , S. Jaber , E. Barbotte , et al., “Impact of Systematic Evaluation of Pain and Agitation in an Intensive Care Unit,” Critical Care Medicine 34, no. 6 (2006): 1691–1699. 16625136 10.1097/01.CCM.0000218416.62457.56

[5] J. W. Devlin , Y. Skrobik , C. Gelinas , et al., “Clinical Practice Guidelines for the Prevention and Management of Pain, Agitation/Sedation, Delirium, Immobility, and Sleep Disruption in Adult Patients in the ICU,” Critical Care Medicine 46, no. 9 (2018): e825–e873. 30113379 10.1097/CCM.0000000000003299

[6] L. E. Vaurio , L. P. Sands , Y. Wang , E. A. Mullen , and J. M. Leung , “Postoperative Delirium: The Importance of Pain and Pain Management,” Anesthesia and Analgesia 102, no. 4 (2006): 1267–1273. 16551935 10.1213/01.ane.0000199156.59226.af

[7] A. De Jong , N. Molinari , S. De Lattre , et al., “Decreasing Severe Pain and Serious Adverse Events While Moving Intensive Care Unit Patients: A Prospective Interventional Study (The NURSE‐DO Project),” Critical Care 17, no. 2 (2013): R74. 23597243 10.1186/cc12683 PMC3672726

[8] A. Yamashita , M. Yamasaki , H. Matsuyama , and F. Amaya , “Risk Factors and Prognosis of Pain Events During Mechanical Ventilation: A Retrospective Study,” Journal of Intensive Care 5 (2017): 17. 28194277 10.1186/s40560-017-0212-5 PMC5299760

[9] M. Choinière , J. Watt‐Watson , J. C. Victor , et al., “Prevalence of and Risk Factors for Persistent Postoperative Nonanginal Pain After Cardiac Surgery: A 2‐Year Prospective Multicentre Study,” Canadian Medical Association Journal 186, no. 7 (2014): E213–E223. 24566643 10.1503/cmaj.131012 PMC3986330

[10] M. E. Koster‐Brouwer , M. Rijsdijk , W. K. M. van Os , et al., “Occurrence and Risk Factors of Chronic Pain After Critical Illness,” Critical Care Medicine 48, no. 5 (2020): 680–687. 32039992 10.1097/CCM.0000000000004259

[11] N. Smaisim , M. Rijsdijk , Y. van der Does , and A. J. C. Slooter , “Pain and Psychopathology After Intensive Care Unit Admission,” Anaesthesia and Intensive Care 52, no. 4 (2024): 232–240. 38879797 10.1177/0310057X241226716 PMC11290044

[12] C. Gelinas and C. Johnston , “Pain Assessment in the Critically Ill Ventilated Adult: Validation of the Critical‐Care Pain Observation Tool and Physiologic Indicators,” Clinical Journal of Pain 23, no. 6 (2007): 497–505. 17575489 10.1097/AJP.0b013e31806a23fb

[13] E. W. Ely , B. Truman , A. Shintani , et al., “Monitoring Sedation Status Over Time in ICU Patients: Reliability and Validity of the Richmond Agitation‐Sedation Scale (RASS),” JAMA 289, no. 22 (2003): 2983–2991. 12799407 10.1001/jama.289.22.2983

[14] N. Bergeron , M. J. Dubois , M. Dumont , S. Dial , and Y. Skrobik , “Intensive Care Delirium Screening Checklist: Evaluation of a New Screening Tool,” Intensive Care Medicine 27, no. 5 (2001): 859–864. 11430542 10.1007/s001340100909

[15] B. F. Olsen , B. T. Valeberg , M. Jacobsen , M. C. Småstuen , K. Puntillo , and T. Rustøen , “Pain in Intensive Care Unit Patients‐A Longitudinal Study,” Nursing Open 8, no. 1 (2021): 224–231. 33318830 10.1002/nop2.621 PMC7729640

[16] S. Nisula , K. M. Kaukonen , S. T. Vaara , et al., “Incidence, Risk Factors and 90‐Day Mortality of Patients With Acute Kidney Injury in Finnish Intensive Care Units: The FINNAKI Study,” Intensive Care Medicine 39, no. 3 (2013): 420–428. 23291734 10.1007/s00134-012-2796-5

[17] G. Chanques , M. Sebbane , E. Barbotte , E. Viel , J. J. Eledjam , and S. Jaber , “A Prospective Study of Pain at Rest: Incidence and Characteristics of an Unrecognized Symptom in Surgical and Trauma Versus Medical Intensive Care Unit Patients,” Anesthesiology 107, no. 5 (2007): 858–860. 18073576 10.1097/01.anes.0000287211.98642.51

[18] R. L. M. van Boekel , M. C. Warlé , R. G. C. Nielen , et al., “Relationship Between Postoperative Pain and Overall 30‐Day Complications in a Broad Surgical Population: An Observational Study,” Annals of Surgery 269, no. 5 (2019): 856–865. 29135493 10.1097/SLA.0000000000002583

[19] F. Coluzzi , F. Bifulco , A. Cuomo , et al., “The Challenge of Perioperative Pain Management in Opioid‐Tolerant Patients,” Therapeutics and Clinical Risk Management 13 (2017): 1163–1173. 28919771 10.2147/TCRM.S141332 PMC5592950

[20] T. Vasilopoulos , R. Wardhan , P. Rashidi , et al., “Patient and Procedural Determinants of Postoperative Pain Trajectories,” Anesthesiology 134, no. 3 (2021): 421–434. 33449996 10.1097/ALN.0000000000003681 PMC8726009

[21] J. A. J. Martyn , J. Mao , and E. A. Bittner , “Opioid Tolerance in Critical Illness,” New England Journal of Medicine 380, no. 4 (2019): 365–378. 30673555 10.1056/NEJMra1800222 PMC6897318

[22] C. Gélinas , “Management of Pain in Cardiac Surgery ICU Patients: Have We Improved Over Time?,” Intensive & Critical Care Nursing 23, no. 5 (2007): 298–303. 17448662 10.1016/j.iccn.2007.03.002

[23] M. Modanloo , A. Mohsenpour , H. Rahmani , et al., “Impact of Implementing the Critical Care Pain Observation Tool on Nurses' Performance in Assessing and Managing Pain in the Critically Ill Patients,” Indian Journal of Critical Care Medicine 23, no. 4 (2019): 165–169. 31130786 10.5005/jp-journals-10071-23146 PMC6521825

[24] J. A. J. Martyn , J. Mao , and E. A. Bittner , “Opioid Tolerance in Critical Illness. Reply,” New England Journal of Medicine 380, no. 16 (2019): e26. 10.1056/NEJMc1902646 30995390

[25] M. M. H. Yang , R. L. Hartley , A. A. Leung , et al., “Preoperative Predictors of Poor Acute Postoperative Pain Control: A Systematic Review and Meta‐Analysis,” BMJ Open 9, no. 4 (2019): e025091. 10.1136/bmjopen-2018-025091 PMC6500309 30940757

[26] E. J. Bartley and R. B. Fillingim , “Sex Differences in Pain: A Brief Review of Clinical and Experimental Findings,” British Journal of Anaesthesia 111, no. 1 (2013): 52–58. 23794645 10.1093/bja/aet127 PMC3690315

[27] J. P. van de Leur , C. P. van der Schans , B. G. Loef , B. G. Deelman , J. H. B. Geertzen , and J. H. Zwaveling , “Discomfort and Factual Recollection in Intensive Care Unit Patients,” Critical Care 8, no. 6 (2004): R467–R473. 15566593 10.1186/cc2976 PMC1065072

[28] S. M. Jakob , E. Ruokonen , R. M. Grounds , et al., “Dexmedetomidine vs. Midazolam or Propofol for Sedation During Prolonged Mechanical Ventilation: Two Randomized Controlled Trials,” JAMA 307, no. 11 (2012): 1151–1160. 22436955 10.1001/jama.2012.304

[29] M. A. Frölich , K. Zhang , and T. J. Ness , “Effect of Sedation on Pain Perception,” Anesthesiology 118, no. 3 (2013): 611–621. 23314164 10.1097/ALN.0b013e318281592d PMC3744342

[30] L. Jessen Lundorf , H. Korvenius Nedergaard , and A. M. Møller , “Perioperative Dexmedetomidine for Acute Pain After Abdominal Surgery in Adults,” Cochrane Database of Systematic Reviews 2, no. 2 (2016): Cd010358. 26889627 10.1002/14651858.CD010358.pub2 PMC10521028

[31] B. L. Erstad and A. E. Patanwala , “Ketamine for Analgosedation in Critically Ill Patients,” Journal of Critical Care 35 (2016): 145–149. 27481750 10.1016/j.jcrc.2016.05.016

[32] J. F. Payen , G. Chanques , J. Mantz , et al., “Current Practices in Sedation and Analgesia for Mechanically Ventilated Critically Ill Patients: A Prospective Multicenter Patient‐Based Study,” Anesthesiology 106, no. 4 (2007): 687–695. 17413906 10.1097/01.anes.0000264747.09017.da

[33] W. B. Cammarano , J. F. Pittet , S. Weitz , R. M. Schlobohm , and J. D. Marks , “Acute Withdrawal Syndrome Related to the Administration of Analgesic and Sedative Medications in Adult Intensive Care Unit Patients,” Critical Care Medicine 26, no. 4 (1998): 676–684. 9559604 10.1097/00003246-199804000-00015

[34] A. C. Faust , P. Rajan , L. A. Sheperd , C. A. Alvarez , P. McCorstin , and R. L. Doebele , “Impact of an Analgesia‐Based Sedation Protocol on Mechanically Ventilated Patients in a Medical Intensive Care Unit,” Anesthesia and Analgesia 123, no. 4 (2016): 903–909. 27644010 10.1213/ANE.0000000000001393 PMC5237378

[35] E. von Oelreich , M. Eriksson , K. F. Sjölund , A. Discacciati , E. Larsson , and A. Oldner , “Opioid Use After Intensive Care: A Nationwide Cohort Study,” Critical Care Medicine 49, no. 3 (2021): 462–471. 33512940 10.1097/CCM.0000000000004896

[36] J. L. Vincent , Y. Shehabi , T. S. Walsh , et al., “Comfort and Patient‐Centred Care Without Excessive Sedation: The eCASH Concept,” Intensive Care Medicine 42, no. 6 (2016): 962–971. 27075762 10.1007/s00134-016-4297-4 PMC4846689

[37] B. S. Smith , D. Yogaratnam , K. E. Levasseur‐Franklin , et al., “Introduction to Drug Pharmacokinetics in the Critically Ill Patient,” Chest 141, no. 5 (2012): 1327–1336. 22553267 10.1378/chest.11-1396

[38] S. Schulz‐Stübner , A. Boezaart , and J. S. Hata , “Regional Analgesia in the Critically Ill,” Critical Care Medicine 33, no. 6 (2005): 1400–1407. 15942362 10.1097/01.ccm.0000165843.39713.ae

[39] M. F. Nordness , C. J. Hayhurst , and P. Pandharipande , “Current Perspectives on the Assessment and Management of Pain in the Intensive Care Unit,” Journal of Pain Research 14 (2021): 1733–1744. 34163231 10.2147/JPR.S256406 PMC8214553

[40] H. Wøien , “Movements and Trends in Intensive Care Pain Treatment and Sedation: What Matters to the Patient?,” Journal of Clinical Nursing 29, no. 7–8 (2020): 1129–1140. 31904888 10.1111/jocn.15179

[41] M. J. Roos‐Blom , D. Dongelmans , W. Stilma , J. J. Spijkstra , E. de Jonge , and N. de Keizer , “Association Between Organizational Characteristics and Adequate Pain Management at the Intensive Care Unit,” Journal of Critical Care 56 (2020): 1–5. 31765909 10.1016/j.jcrc.2019.11.010

