# Supplementary material for: Acute Pain During Intensive Care: Prevalence, Predisposing Factors and Pain Trajectories. A Prospective Multicenter Observational Study
Source: Acta Anaesthesiol Scand. 2026 Aug 24;70(9):e70322. doi: 10.1111/aas.70322 (PMC13503037; doi:10.1111/aas.70322)
Supplement: Supplementary file 1 [file AAS-70-0-s001.docx]

**SUPPLEMENTARY MATERIAL**

**Acute pain during intensive care: prevalence, predisposing factors and pain trajectories. A prospective multicenter observational study**

**Supporting information: opioid conversion ratios and pain related variables**

**Table of Content**

Table S1	3

Table S2	4

Table S3	5

Table S4	5

Table S5A	6

Table S5B	7

Table S6	8

Table S7	9

Table S8	10

Table S9A	11

Table S9B	12

Table S9C	13

Table S10	14

Figure S1	15

Figure S2	16

Figure S3	17

Figure S4	18

Figure S5	19

Figure S6	20

Figure S7	21

Figure S8	22

**Table S1 Opioid conversion ratios**

| **Opioid, cumulative daily dose, *mg*** | **Admission route** | **Conversion ratio to peroral morphine, *mg*** | **Reference** |
| --- | --- | --- | --- |
| Alfentanil | intravenous | 1:30 | [1] |
| Buprenorphine | Intravenous/  transdermal | 1:110 | [2] |
|  | sublingual | 1:50 | [3] |
| Fentanyl | Intravenous/  transdermal | 1:70 | [4] |
|  | sublingual | 1:50 | [5] |
| Codeine | peroral | 1:0.1 | [3] |
| Methadone | intravenous | 1:13.5 | [6] |
|  | peroral | 1:8 | [7] |
| Morphine | intravenous | 1:3 | [4] |
|  | peroral | 1:1 | - |
| Oxycodone | intravenous | 1:3 | [4] |
|  | peroral | 1:1.5 |  |
|  | intramuscular | 1:3 |  |
| Remifentanil | intravenous | 1:1200 | [8] |
| Sufentanil | intravenous | 1:2000 | [1] |
| Tramadol | intravenous | 1:0.1 | [9] |
|  | peroral | 1:0.1 |  |

**References:**

1.	Coda, B.A., *Opioids*, in *Clinical Anesthesia*. 2006, Wolters Kluwer PE. p. 353-383.

2.	Sittl, R., R. Likar, and B.P. Nautrup, Equipotent doses of transdermal fentanyl and transdermal buprenorphine in patients with cancer and noncancer pain: results of a retrospective cohort study. *Clin Ther*, 2005. **27**(2):225-37

3.	Svendsen, K., P. Borchgrevink, O. Fredheim, et al., Choosing the unit of measurement counts: the use of oral morphine equivalents in studies of opioid consumption is a useful addition to defined daily doses. *Palliat Med*, 2011. **25**(7):725-32

4.	Treillet, E., S. Laurent, and Y. Hadjiat, Practical management of opioid rotation and equianalgesia. *J Pain Res*, 2018. **11**:2587-2601

5.	Shimoyama, N., I. Gomyo, O. Teramoto, et al., Efficacy and safety of sublingual fentanyl orally disintegrating tablet at doses determined from oral morphine rescue doses in the treatment of breakthrough cancer pain. *Jpn J Clin Oncol*, 2015. **45**(2):189-96

6.	Walker, P.W., S. Palla, B.L. Pei, et al., Switching from methadone to a different opioid: what is the equianalgesic dose ratio? *J Palliat Med*, 2008. **11**(8):1103-8

7.	Lawlor, P.G., K.S. Turner, J. Hanson, et al., Dose ratio between morphine and methadone in patients with cancer pain: a retrospective study. *Cancer*, 1998. **82**(6):1167-73

8.	Glass, P.S., I.A. Iselin-Chaves, D. Goodman, et al., Determination of the potency of remifentanil compared with alfentanil using ventilatory depression as the measure of opioid effect. *Anesthesiology*, 1999. **90**(6):1556-63

9.	Wilder-Smith, C.H., J. Schimke, B. Osterwalder, et al., Oral tramadol, a mu-opioid agonist and monoamine reuptake-blocker, and morphine for strong cancer-related pain. *Ann Oncol*, 1994. **5**(2):141-6

**Table S2 Pain medication used for chronic pain in the study cohort before ICU admission.**

| Pain medication at admission | *n* (%) |
| --- | --- |
| Weak opioid^a^, | 40 (5.6) |
| Strong opioid^b^, | 30 (4.2) |
| Antiepileptic medication^c^, | 38 (5.3) |
| Antidepressants^d^, | 16 (2.3) |
| Paracetamol, | 65 (9.1) |
| NSAID, | 32 (4.5) |
| Nonpharmacological treatment^e^, | 12 (1.7) |
| No pain treatment, | 195 (27.4) |
| Information not available, | 9 (1.3) |

NSAID -Nonsteroidal anti-inflammatory drugs

^a^ Regular use of weak prescription opioid (tramadol, codeine) or moderate opioid buprenorphine prior to ICU admission.

^b^ Regular use of strong prescription opioids (fentanyl, oxycodone, morphine, methadone) prior to ICU admission.

^c^ Regular use of antiepileptics (pregabalin, gabapentin, carbamazepine, oxcarbazepine, lamotrigine) prescribed for pain management prior to ICU admission.

^d^ Regular use of serotonin-norepinephrine reuptake inhibitors (duloxetine, venlafaxine) or tricyclic antidepressants (amitriptyline, nortriptyline) prescribed for pain management prior to ICU admission.

^e^ Physiotherapy, transcutaneous electrical nerve stimulation (TENS), warm or cold dressings, spinal cord stimulation, repetitive transcranial magnetic stimulation (rTMS), psychological interventions, complementary or alternative medicine used for pain management prior to ICU admission.

**Table S3 ercentage values of the daily distribution of pain in ICU patients by evaluation day.**

|  | **Day** | | | | | | | | | | | | | |
| --- | --- | --- | --- | --- | --- | --- | --- | --- | --- | --- | --- | --- | --- | --- |
| **Pain state** | 1 | 2 | 3 | 4 | 5 | 6 | 7 | 8 | 9 | 10 | 11 | 12 | 13 | 14 |
| Pain data missing | 89 (12.5%) | 15 (2.1%) | 22 (5.0%) | 13 (4.5%) | 17 (7.5%) | 10 (5.6%) | 10 (6.4%) | 14 (10.4%) | 5 (4.5%) | 10 (10.1%) | 4 (4.8%) | 5 (7.1%) | 7 (10.9%) | 19 (32.2%) |
| Less than moderate pain | 350 (49.2%) | 299 (42.8%) | 209 (47.4%) | 157 (54.1%) | 103 (45.6%) | 75 (42.4%) | 78 (49.7%) | 63 (46.7%) | 49 (44.1%) | 49 (49.5%) | 43 (51.8%) | 33 (47.1%) | 35 (54.7%) | 26 (44.1%) |
| At least moderate pain | 272 (38.3%) | 384 (55.0%) | 210 (47.6%) | 120 (41.4%) | 106 (46.9%) | 92 (52.0%) | 69 (43.9%) | 58 (43.0%) | 57 (51.4%) | 40 (40.4%) | 36 (43.4%) | 32 (45.7%) | 22 (34.4%) | 14 (23.7%) |

**Table S4** **Daily opioid doses converted to oral morphine equivalents.**

| **Subgroup** |  | **Day 1**  **n=711** | **Day 2**  **n=698** | **Day 3**  **n=441** | **Day 4**  **n=290** | **Day 5**  **n=226** | **Day 6**  **n=177** | **Day 7**  **n=157** | **Day 8**  **n=135** | **Day 9**  **n=111** | **Day 10**  **n=99** | **Day 11**  **n=83** | **Day 12**  **n=70** | **Day 13**  **n=64** | **Last day**  **n=59** |
| --- | --- | --- | --- | --- | --- | --- | --- | --- | --- | --- | --- | --- | --- | --- | --- |
| Surgical | Median | 27 | 48 | 34.5 | 45 | 48.5 | 52.5 | 45 | 42.8 | 55.5 | 50.3 | 43.5 | 47.5 | 33 | 6 |
|  | Min | 0 | 0 | 0 | 0 | 0 | 0 | 0 | 0 | 0 | 0 | 0 | 0 | 0 | 0 |
|  | Max | 1460 | 1275 | 1110.0 | 885.5 | 1960 | 302 | 993.5 | 829 | 1242.5 | 1508.5 | 1960 | 883 | 1581 | 912 |
| Non-surgical | Median | 17.5 | 36.8 | 30 | 33 | 39 | 44.3 | 33 | 40.5 | 45 | 37.0 | 33 | 25.5 | 22.5 | 7.5 |
|  | Min | 0 | 0 | 0 | 0 | 0 | 0 | 0 | 0 | 0 | 0 | 0 | 0 | 0 | 0 |
|  | Max | 186 | 385 | 332.5 | 265.5 | 387 | 369.5 | 343.4 | 636 | 266 | 378 | 750 | 474 | 297 | 154 |

n= number of study patients present in the ICU on each day of follow up.

**Table S5A Features of non-opioid pain alleviation during treatment in the ICU.**

|  | | **Day 1**  **n=711** | **Day 2**  **n=698** | **Day 3**  **n=441** | **Day 4**  **n=290** | **Day 5**  **n=226** | **Day 6**  **n=177** | **Day 7**  **n=157** | **Day 8**  **n=135** | **Day 9**  **n=111** | **Day 10**  **n=99** | **Day 11**  **n=83** | **Day 12**  **n=70** | **Day 13**  **n=64** | **Last day**  **n=59** |
| --- | --- | --- | --- | --- | --- | --- | --- | --- | --- | --- | --- | --- | --- | --- | --- |
| Non-opioid pain medication | Paracetamol, *n* (%) | 256  (36.0) | 386  (55.3) | 228  (51.7) | 148  (51) | 116  (51.3) | 78  (44) | 86  (54.8) | 70  (51.9) | 54  (48.6) | 46  (46.5) | 35  (42.2) | 35  (50) | 30  (46.9) | 22  (37.3) |
|  | NSAIDs, *n* (%) | 5  (0.7) | 15  (2.1) | 15  (3.4) | 9  (3.1) | 7  (3.1) | 3  (3.1) | 3  (1.9) | 4  (3) | 1  (0.9) | 1  (1) | 1  (1.2) | 0 | 0 | 1  (1.7) |
|  | S-Ketamine^a^, *n* (%) | 36  (5.1) | 49  (7) | 31  (7) | 21  (7.2) | 16  (7.1) | 13  (7.3) | 10  (6.3) | 9  (6.7) | 8  (7.2) | 9  (9.1) | 8  (9.6) | 8  (11.4) | 5  (7.8) | 2  (3.3) |
|  | Gabapentinoids^b^, *n* (%) | 31  (4.3) | 76  (10.9) | 54  (12.2) | 29  (10) | 22  (9.7) | 16  (9) | 20  (12.7) | 21  (15.6) | 18  (16.2) | 6  (6.1) | 16  (19.3) | 13  (18.6) | 13  (20.3) | 11  (16.9) |
|  | SNRIs or other neuropathic  pain medications^c^, *n* (%) | 2  (0.3) | 2  (0.3) | 3  (0.7) | 2  (0.7) | 2  (0.9) | 2  (1.2) | 1  (0.6) | 1  (0.7) | 1  (0.9) | 1  (1.0) | 1  (1.2) | 2  (2.9) | 2  (3.1) | 2  (3.4) |
| Local analgesia | Neuraxial analgesia^d^,  *n* (%) | 153  (21.4) | 159  (22.8) | 94  (21.3) | 55  (19.0) | 44  (19.4) | 31  (17.5) | 24  (15.3) | 22  (16.3) | 21  (18.9) | 15  (15.2) | 9  (10.8) | 8  (11.4) | 7  (10.9) | 4  (6.8) |
|  | Peripheral  nerve block, *n* (%) | 13  (1.8) | 10  (1.4) | 10  (2.3) | 7  (2.4) | 7  (3.1) | 5  (2.8) | 5  (3.2) | 4  (3.0) | 3  (2.7) | 2  (2) | 1  (1.2) | 2  (2.9) | 1  (1.6) | 0 |

  Data are presented in number of patients (*n*) and their proportion in percentages in the cohort of patients treated during the day of follow-up day or in median and range (min – max). ICU = Intensive Care Unit, NSAID = Non-Steroidal Anti-Inflammatory Drug

^a^ Oral or intravenous S-ketamine.

^b^ Gabapentinoids used for pain management: gabapentin, pregabalin.

^c^ Serotonin-norepinephrine reuptake inhibitors (duloxetine, venlafaxine), tricyclic antidepressants or other antiepileptic medications administered for pain relief.

^d^ Neuraxial anesthesia: epidural block or continuous infusion, spinal block.

**Table S5B Number of ICU patients receiving non-opioid medication categorized by surgical and non-surgical patient groups.**

| **Non-opioid medication** | **Subgroup** | **Day 1**  **n=711** | **Day 2**  **n=698** | **Day 3**  **n=441** | **Day 4**  **n=290** | **Day 5**  **n=226** | **Day 6**  **n=177** | **Day 7**  **n=157** | **Day 8**  **n=135** | **Day 9**  **n=111** | **Day 10**  **n=99** | **Day 11**  **n=83** | **Day 12**  **n=70** | **Day 13**  **n=64** | **Last day**  **n=59** |
| --- | --- | --- | --- | --- | --- | --- | --- | --- | --- | --- | --- | --- | --- | --- | --- |
| Paracetamol | Surgical | 185 | 281 | 146 | 91 | 71 | 48 | 52 | 41 | 31 | 25 | 18 | 17 | 15 | 13 |
|  | Non-surgical | 71 | 105 | 82 | 57 | 45 | 33 | 34 | 29 | 23 | 21 | 17 | 18 | 15 | 9 |
| NSAIDs | Surgical | 4 | 12 | 14 | 7 | 6 | 3 | 3 | 3 | 1 | 1 | 1 | 0 | 0 | 1 |
|  | Non-surgical | 1 | 3 | 1 | 2 | 1 | 0 | 0 | 1 | 0 | 0 | 0 | 0 | 0 | 0 |
| S-Ketamine^a^ | Surgical | 17 | 31 | 17 | 12 | 12 | 7 | 4 | 4 | 3 | 4 | 2 | 2 | 2 | 1 |
|  | Non-surgical | 19 | 18 | 14 | 9 | 4 | 6 | 6 | 5 | 5 | 5 | 6 | 6 | 3 | 1 |
| Gabapentinoids^b^ | Surgical | 19 | 61 | 40 | 23 | 17 | 13 | 17 | 16 | 12 | 9 | 8 | 6 | 6 | 5 |
|  | Non-surgical | 12 | 15 | 14 | 6 | 5 | 3 | 3 | 5 | 6 | 7 | 8 | 7 | 7 | 6 |
| SNRIs or other neuropathic pain medications^c^ | Surgical | 2 | 1 | 2 | 0 | 0 | 1 | 0 | 0 | 0 | 0 | 0 | 0 | 0 | 0 |
|  | Non-surgical | 0 | 1 | 1 | 2 | 2 | 1 | 1 | 1 | 1 | 1 | 1 | 2 | 2 | 2 |

n= number of study patients present in the ICU on each day of follow up.

ICU = Intensive Care Unit, NSAID = Non-Steroidal Anti-Inflammatory Drug

^a^ Oral or intravenous S-ketamine

^b^ Gabapentinoids used for pain management: gabapentin, pregabalin

^c^ Serotonin-norepinephrine reuptake inhibitors (duloxetine, venlafaxine), tricyclic antidepressants or other antiepileptic medications administered for pain relief

**Table S6 Use of continuous sedation and neuromuscular blocking agents, level of sedation and prevalence of delirium during treatment in the ICU.**

|  | **Day 1**  **n=711** | **Day 2**  **n=698** | **Day 3**  **n=441** | **Day 4**  **n=290** | **Day 5**  **n=226** | **Day 6**  **n=177** | **Day 7**  **n=157** | **Day 8**  **n=135** | **Day 9**  **n=111** | **Day 10**  **n=99** | **Day 11**  **n=83** | **Day 12**  **n=70** | **Day 13**  **n=64** | **Last day**  **n=59** |
| --- | --- | --- | --- | --- | --- | --- | --- | --- | --- | --- | --- | --- | --- | --- |
| Sedation^a^, *n* (%) | 267  (37.6) | 247  (35.4) | 164  (37.2) | 117  (40.3) | 100  (44.2) | 80  (45.2) | 69  (43.9) | 62  (45.9) | 51  (45.9) | 40  (40.4) | 37  (44.6) | 31  (44.3) | 26  (40.6) | 6  (10.2) |
| NMBA^b^, *n* (%) | 69  (9.7) | 50  (7.1) | 33  (7.5) | 18  (6.2) | 7  (3.1) | 10  (5.6) | 5  (3.2) | 7  (5.2) | 7  (6.3) | 6  (6) | 2  (2.4) | 3  (4.3) | 4  (6.3) | 1  (1.7) |
| RASS max, median (range) | 0  (-5 – 3) | 0  (-5 – 3) | 0  (-5 – 3) | 0  (-5 – 4) | 0  (-5 – 4) | 0  (-5–3) | 0  (-5 – 1) | 0  (-5–4) | 0  (-5–3) | 0  (-5–3) | 0  (-5–4) | 0  (-3–3) | 0  (-3–3) | 0  (-5–2) |
| RASS min,  median (range) | -3  (-5 – 3) | -1  (-5 – 1) | -1  (-5–2) | -1  (-5 – 1) | -1  (-5 – 1) | -2   (-5–4) | -1  (-5 (-1)) | -2  (-5 – 1) | -1  (-5 – 1) | -1  (-5 – 1) | -1   (-5 – 1) | -1  (-5 – 1) | -1  (-5–0) | -1  (-5–0) |
| Delirium^c^, *n* (%) | 23  (3.2) | 48  (6.9) | 58  (13.2) | 47  (16.2) | 38  (16.8) | 39  (22) | 38  (24.2) | 32  (23.7) | 26  (23.4) | 26  (26.2) | 22  (26.5) | 18  (25.7) | 18  (28.1) | 12  (20.3) |

Data are presented in number of patients (*n*) and their proportion in percentages in the cohort of patients treated during the day of follow-up day or in median and range (min – max). ICU = Intensive Care Unit, RASS = Richmond Agitation-Sedation Scale

^a^ Sedation ≥6 h infusion: propofol, dexmedetomidine, midazolam.

^b^ Neuromuscular blocking agent (NMBA): rocuronium, cis-atracurium.

^c^ Delirium defined as ICDSC ≥ 4 (Intensive Care Delirium Screening Checklist).

**Table S7 Medical devices patients had on each day of follow-up in the ICU.**

| **Medical devices** | **Day 1 n, (%)** | **Day 2 n, (%)** | **Day 3 n, (%)** | **Day 4 n, (%)** | **Day 5 n, (%)** | **Day 6 n, (%)** | **Day 7 n, (%)** | **Day 8 n, (%)** | **Day 9 n, (%)** | **Day 10 n, (%)** | **Day 11 n, (%)** | **Day 12 n, (%)** | **Day 13 n, (%)** | **Last day n, (%)** |
| --- | --- | --- | --- | --- | --- | --- | --- | --- | --- | --- | --- | --- | --- | --- |
| Central IV line^a^ | 449  (63.2) | 466  (66.8) | 315  (71.4) | 229  (79) | 191  (84.5) | 160  (90.4) | 143  (91.1) | 126  (93.3) | 106  (95.5) | 96  (97) | 79  (95.2) | 65  (92.9) | 59  (92.2) | 45  (76.3) |
| Arterial line | 706  (99.3) | 695  (99.6) | 432  (98) | 287  (99) | 223  (98.7) | 174  (98.3) | 155  (98.7) | 133  (98.5) | 110  (99.1) | 95  (96) | 81  (97.6) | 70  (100) | 61  (95.3) | 50  (84.7) |
| Urinary/ rectal catheter | 700  (98.5) | 687  (98.4) | 430  (97.5) | 285  (98.3) | 222  (98.2) | 174  (98.3) | 154  (98.1) | 135  (100) | 110  (99.1) | 99  (100) | 82  (98.8) | 69  (98.6) | 64  (100) | 55  (93.2) |
| Pleural drain | 101  (14.2) | 116  (16.6) | 57  (12.9) | 42  (14.5) | 35  (15.5) | 24  (13.6) | 23  (14.6) | 24  (17.8) | 22  (19.8) | 18  (18.2) | 15  (18.1) | 16  (22.9) | 14  (21.9) | 6  (10.2) |
| Intubation tube | 407  (57.2) | 220  (31.5) | 145  (32.9) | 113  (39) | 98  (43.4) | 82  (46.3) | 68  (43.3) | 57  (42.2) | 52  (46.8) | 37  (37.4) | 28  (33.7) | 24  (34.3) | 18  (28.1) | 1  (1.7) |
| Tracheostomy tube | 21  (3) | 22  (3.2) | 21  (4.8) | 16  (5.5) | 14  (6.2) | 14  (7.9) | 12  (7.6) | 11  (8.1) | 8  (7.2) | 5  (5.1) | 8  (9.6) | 7  (10) | 10  (15.6) | 9  (15.3) |
| Plastering | 16  (2.3) | 16  (2.3) | 20  (4.5) | 11  (3.8) | 9  (4) | 4  (2.3) | 6  (3.8) | 4  (3) | 3  (2.7) | 3  (3) | 2  (2.4) | 2  (2.9) | 1  (1.6) | 2  (3.4) |
| Surgical side drains | 309  (43.5) | 302  (43.3) | 154  (34.9) | 89  (30.7) | 62  (27.4) | 39  (22) | 33  (21) | 29  (21.5) | 22  (19.8) | 23  (23.2) | 15  (18.1) | 14  (20) | 14  (21.9) | 15  (25.4) |
| Nasal gastric tube/ feeding tube | 419  (58.9) | 397  (56.9) | 266  (60.3) | 199  (68.6) | 163  (72.1) | 140  (79.1) | 129  (82.2) | 115  (85.2) | 97  (87.4) | 84  (84.8) | 70  (84.3) | 60  (85.7) | 54  (84.4) | 39  (66.1) |
| Cooling devices^b^ | 21  (3) | 21  (3) | 21  (4.8) | 11  (3.8) | 3  (3.1) | 2  (1.1) | 2  (1.3) | 2  (1.5) | 2  (1.8) | 2  (2) | 2  (2.4) | 3  (4.3) | 1  (1.6) | 0  (0) |
| Collar | 6  (0.8) | 4  (0.6) | 2  (0.5) | 3  (1) | 1  (0.4) | 1  (0.6) | 1  (0.6) | 1  (0.7) | 1  (0.9) | 1  (1) | 1  (1.2) | 1  (1.4) | 1  (1.6) | 0  (0) |
| Physical restraint | 47  (6.6) | 33  (4.7) | 28  (6.3) | 20  (6.9) | 26  (11.5) | 22  (12.4) | 15  (9.6) | 12  (8.9) | 13  (11.7) | 12  (12.1) | 8  (9.6) | 6  (8.6) | 7  (10.9) | 3  (5.1) |

^a^ Central venous catheter, dialysis catheter, pulmonary artery catheter.

^b^ External and internal cooling devices.

**Table S8 Procedures performed during follow-up in the ICU and number of complications.**

| **Procedures** | **Day 1 n, (%)** | **Day 2 n, (%)** | **Day 3 n, (%)** | **Day 4 n, (%)** | **Day 5 n, (%)** | **Day 6 n, (%)** | **Day 7 n, (%)** | **Day 8 n, (%)** | **Day 9 n, (%)** | **Day 10 n, (%)** | **Day 11 n, (%)** | **Day 12 n, (%)** | **Day 13 n, (%)** | **Last day n, (%)** |
| --- | --- | --- | --- | --- | --- | --- | --- | --- | --- | --- | --- | --- | --- | --- |
| Central IV-line insertion | 334 (47) | 55 (7.9) | 19 (4.3) | 17 (5.9) | 4 (1.8) | 8 (4.5) | 0 (0) | 9 (6.7) | 2 (1.8) | 3, (3) | 1, (1.2) | 2, (2.9) | 4, (6.3) | 2, (3.4) |
| Arterial line insertion | 478 (67.2) | 26 (3.7) | 18 (4.1) | 9 (3.1) | 15 (6.6) | 7 (4) | 8 (5.1) | 5 (3.7) | 7 (6.3) | 7 (7.1) | 2 (2.4) | 3 (4.3) | 5 (7.8) | 1 (1.7) |
| Urinary/ rectal catheter insertion | 379 (53.3) | 25 (3.6) | 19 (4.3) | 7 (2.4) | 10 (4.4) | 7 (4) | 6 (3.8) | 7 (5.2) | 5 (4.5) | 2 (2) | 1 (1.2) | 6 (8.6) | 5 (7.8) | 1 (1.7) |
| Pleural drain insertion/ removal | 82 (11.5) | 10 (1.4) | 3 (0.7) | 5 (1.7) | 2 (0.9) | 1 (0.6) | 5 (3.2) | 5 (3.7) | 2 (1.8) | 0 (0) | 3 (3.6) | 1 (1.4) | 1 (1.6) | 0 (0) |
| Endoscopy | 50 (7) | 32 (4.6) | 25 (5.7) | 17 (5.9) | 25 (11.1) | 17 (9.6) | 15 (9.6) | 15 (11.1) | 6 (5.4) | 10 (10.1) | 9 (10.8) | 6 (8.6) | 10 (15.6) | 2(3.4) |
| Suction | 247 (34.7) | 167 (23.9) | 135 (30.6) | 110 (37.9) | 94 (41.6) | 83 (46.9) | 69 (43.9) | 56 (41.5) | 50 (45) | 35 (35.4) | 33 (39.8) | 30 (42.9) | 21 (32.8) | 8 (13.6) |
| Plastering | 7 (1) | 1 (0.1) | 1(0.2) | 0 (0) | 3 (1.3) | 2 (1.1) | 1 (0.6) | 0 (0) | 1 (0.9) | 0 (0) | 0 (0) | 0 (0) | 0 (0) | 0 (0) |
| IV cannula insertion | 316 (44.4) | 37 (5.3) | 21 (4.8) | 16 (5.5) | 18 (8) | 4 (2.3) | 8 (5.1) | 11 (8.1) | 3 (2.7) | 4 (4) | 5 (6) | 1 (1.4) | 5 (7.8) | 2 (3.4) |
| Lumbar/epidural/  spinal punction | 51 (7.2) | 13 (1.9) | 4 (0.9) | 4 (1.4) | 3 (1.3) | 0 (0) | 0 (0) | 0 (0) | 1 (0.9) | 0 (0) | 1 (1.2) | 0 (0) | 0 (0) | 0 (0) |
| Wound care | 64 (9) | 143 (20.5) | 119 (27) | 77 (26.6) | 62 (27.4) | 50 (28.2) | 39 (24.8) | 38 (28.1) | 31 (27.9) | 28 (28.3) | 23 (27.7) | 19 (27.1) | 19 (29.7) | 18 (30.5) |
| Tracheostomy | 14 (2) | 2 (0.3) | 3 (0.7) | 0 (0) | 1 (0.4) | 1 (0.6) | 1 (0.6) | 1 (0.7) | 1 (0.9) | 2 (2) | 2 (2.4) | 0 (0) | 4 (6.3) | 0 (0) |
| Surgery^a^ | 314 (44.2) | 33 (4.7) | 21 (4.8) | 17 (5.9) | 8 (3.5) | 9 (5.1) | 5 (3.2) | 8 (5.9) | 8 (7.2) | 2 (2) | 1 (1.2) | 4 (5.7) | 7 (10.9) | 0(0) |
| Small procedure^b^ | 39 (5.5) | 41 (5.9) | 31 (7) | 15 (5.2) | 12 (5.3) | 16 (9) | 12 (7.6) | 6 (4.4) | 7 (6.3) | 6 (6.1) | 5 (6) | 6 (8.6) | 3 (4.7) | 3 (5.1) |
| Physiotherapy^c^ | 43 (6) | 257 (36.8) | 172 (39) | 99 (34.1) | 79 (35) | 63 (35.6) | 57 (36.3) | 43 (31.9) | 40 (36) | 34 (34.3) | 35 (42.2) | 31 (44.3) | 25 (39.1) | 32(54.2) |
| Mobilization^d^ | 297 (41.8) | 524 (75.1) | 355 (80.5) | 232 (80) | 196 (86.7) | 148 (83.6) | 138 (87.9) | 120 (88.9) | 94 (84.7) | 86 (86.9) | 71 (85.5) | 68 (97.1) | 57 (89.1) | 45 (76.3) |
| Drain insertion/removal | 173 (24.3) | 66 (9.5) | 30 (6.8) | 33 (11.4) | 21 (9.3) | 7 (4) | 12 (7.6) | 9 (6.7) | 6 (5.4) | 4 (4) | 5 (6) | 6 (8.6) | 3 (4.7) | 0 (0) |
| Defibrillation/cardioversion/  resuscitation | 27 (3.8) | 6 (0.9) | 4 (0.9) | 3 (1) | 1 (0.4) | 0 (0) | 0 (0) | 1 (0.7) | 1 (0.9) | 2 (2) | 0 (0) | 0 (0) | 0 (0) | 0 (0) |
| Dialysis | 20 (2.8) | 42 (6) | 40 (9.1) | 39(13.4) | 33 (14.6) | 26 (14.7) | 26 (16.6) | 20 (14.8) | 19 (17.1) | 20 (20.2) | 17 (20.5) | 18 (25.7) | 15 (23.4) | 5 (8.5) |
| **Complications**^e^ | 26 (4) | 16 (2.3) | 4 (0.9) | 10 (3.4) | 1 (0.4) | 1 (0.6) | 4 (2.5) | 4 (3) | 4 (3.6) | 1 (1) | 4 (4.8) | 2 (2.9) | 3 (4.7) | 0(0) |

^a^ Surgery is defined as a major procedure performed in the operating theatre.

^b^ Smaller procedures are defined as bedside procedures, for example, wound closure.

^c^ Physiotherapy is a daily exercise with physiotherapist or a bedside nurse including breathing exercises, passive and active limb movement as well as sitting, standing and walking practice.

^d^ Mobilization is defined as positioning, turning over, transferring, for example, to CT or MRI suits.

^e^ Complications, meaning unwanted adverse effects from medical procedures, were classified into the following categories: massive bleeding (needed surgical intervention and or blood transfusion), sudden cardiac arrest, repeated punctures doe central line or arterial line insertion, pneumothorax, desaturation (SpO_2_ below 90%), hypotonia (systolic blood pressure below 80 mmHg from other reasons than bleeding, arrythmias (most commonly atrial fibrillation).

**Table S9A Results of the mixed effects logistic regression models of pain at rest during the whole period of follow-up in the ICU. Variables were selected for the adjusted model based on clinical relevance and the results of the univariable model.**

|  | **Unadjusted model** | | **Adjusted model** | |
| --- | --- | --- | --- | --- |
| **Variable** | **OR (95% CI)** | **P-value** | **OR (95% CI)** | **P-value** |
| Age | 0.50 (0.32-0.79) | <0.01 | 1.20 (0.71-2.02) | 0.50 |
| Female gender | 1.46 (1.11-1.90) | <0.01 | 1.58 (1.19-2.11) | <0.01 |
| BMI^a^ | 1.00 (0.97-1.02) | 0.71 | NC | NC |
| No comorbidities^b^ | 0.94 (0.72-1.22) | 0.63 | NC | NC |
| Diabetes^c^ | 1.09 (0.81-1.48) | 0.56 | 1.31 (0.95-1.79) | 0.095 |
| Cancer^d^ | 0.92 (0.69-1.24) | 0.59 | NC | NC |
| Psychiatric disorder^e^ | 1.95 (1.13-3.37) | 0.017 | 1.18 (0.66-2.12) | 0.58 |
| Persistent pain^f^ | 1.50 (1.08-2.08) | 0.015 | NC | NC |
| Nociceptive pain^g^ | 1.41 (0.98-2.04) | 0.067 | 1.00 (0.67-1.48) | 0.98 |
| Neuropathic pain^h^ | 1.92 (1.13-3.26) | 0.016 | 1.34 (0.74-2.42) | 0.34 |
| Opioid use^i^ | 0.82 (0.43-1.57) | 0.55 | 0.90 (0.31-2.57) | 0.84 |
| Surgical diagnosis at admission^j^ | 2.33 (1.80-3.02) | <0.01 | 1.94 (1.42-2.64) | 0.01 |
| SAPS II, per 10 units^k^ | 0.80 (0.74-0.88) | <0.01 | 0.89 (0.80-0.99) | 0.035 |
| Vasopressor^l^ | 0.92 (0.75-1.14) | 0.46 | NC | NC |
| Sedation^m^ | 1.02 (0.84-1.24) | 0.83 | 0.75 (0.60-0.94) | 0.013 |
| Dexmedetomidine | 1.12 (0.86-1.46) | 0.39 | 1.41 (1.02-1.95) | 0.04 |
| MEQ, per 10 units^n^ | 1.18 (1.15-1.21) | <0.01 | 1.20 (1.17-1.23) | <0.01 |
| Regional analgesia^o^ | 1.27 (1.03-1.58) | 0.029 | NC | NC |
| Delirium^p^ | 0.93 (0.72-1.21) | 0.59 | NC | NC |
| Fever^q^ | 1.33 (1.07-1.64) | <0.01 | NC | NC |
| Leukocytosis, per 10 units^r^ | 1.06 (0.90-1.25) | 0.46 | 1.09 (0.92-1.30) | 0.32 |
| CRP, per 10 units^s^ | 1.01 (1.00-1.01) | 0.30 | 1.00 (0.99-1.01) | 0.42 |

NC not calculated

^a^ Body Mass Index in kg/m^2^.

^b^ No diagnosed diseases prior ICU admission.

^c^ Diabetes mellitus diagnosed prior to ICU admission.

^d^ Malignant tumor or leukemia diagnosed prior to ICU admission.

^e^ Psychiatric disease defined as fulfilling ICD10 diagnostic criteria prior to ICU admission.

^f^ Persistent pain (nociceptive, neuropathic, or unspecified) diagnosis prior to ICU admission.

^g^ Persistent pain described as nociceptive (pain arising from actual or threatened non-neural tissue damage) present prior to ICU admission.

^h^ Persistent pain described as neuropathic (neurological lesion or disease and neuroanatomically plausible pain distribution) diagnosed prior to ICU admission.

^i^ Regular use of prescription opioids prior to ICU admission.

^j^ Admission after surgery, postoperative complications, bleeding, perioperative emergencies, pancreatitis, acute liver failure, trauma.

^k^ Simplified Acute Physiology Score II.

^l^ Noradrenalin administered > 0.1 µg/kg/min over 1 hour per calendar day.

^m^ Continuous administration of a sedative agent (propofol, benzodiazepines, α^2^ -agonists, barbiturates, S-ketamine or inhalation agent) for over 6 hours in calendar day in ICU (excluding sedatives administered outside of ICU).

^n^ Opioid dose in MEQ (oral morphine equivalents) administered in ICU per calendar day.

^o^ Local, regional or neuraxial anesthesia administered in ICU per calendar day.

^p^ Intensive Care Delirium Screening Checklist ≥ 4 points during calendar day in ICU

^q^ Body temperature exceeding 38 degrees Celsius at any time during calendar day.

^r^ Leukocyte count > 8.2^9^/l during calendar day in ICU.

^s^ CRP (C Reactive protein) > 100 mg/l during calendar day ICU.

**Table S9B. Results of the mixed effects logistic regression models of pain at rest for the first 3 days of follow-up in the ICU, excluding elective postoperative patients. Variables were selected for the adjusted model based on clinical relevance and the results of the univariable model.**

|  | **Unadjusted model** | | **Adjusted model** | |
| --- | --- | --- | --- | --- |
| **Variable** | **OR (95% CI)** | **P-value** | **OR (95% CI)** | **P-value** |
| Age | 0.47 (0.24-0.94) | 0.034 | 1.62 (0.69-3.78) | 0.27 |
| Female gender | 1.29 (0.82-2.04) | 0.27 | 1.50 (0.93-2.41) | 0.095 |
| BMI ^a^ | 1.01 (0.97-1.05) | 0.61 | NC | NC |
| No comorbidities ^b^ | 0.90 (0.58-1.39) | 0.63 | NC | NC |
| Diabetes ^c^ | 1.25 (0.74-2.11) | 0.40 | 1.46 (0.86-2.47) | 0.16 |
| Cancer ^d^ | 1.01 (0.56-1.84) | 0.97 | NC | NC |
| Psychiatric disorder ^e^ | 2.00 (0.83-4.85) | 0.12 | 1.49 (0.58-3.82) | 0.41 |
| Persistent pain ^f^ | 1.03 (0.56-1.87) | 0.94 | NC | NC |
| Nociceptive pain ^g^ | 0.82 (0.40-1.66) | 0.58 | 0.57 (0.27-1.21) | 0.14 |
| Neuropathic pain ^h^ | 1.78 (0.69-4.60) | 0.23 | 1.74 (0.62-4.86) | 0.29 |
| Opioid use ^i^ | 0.97 (0.32-2.96) | 0.95 | 0.98 (0.25-3.77) | 0.97 |
| Surgical diagnosis at admission ^j^ | 3.82 (2.44-6.00) | <0.01 | 2.68 (1.65-4.33) | <0.01 |
| SAPS II, per 10 units ^k^ | 0.75 (0.65-0.87) | <0.01 | 0.83 (0.69-1.00) | 0.053 |
| Vasopressor ^l^ | 0.88 (0.59-1.31) | 0.52 | NC | NC |
| Sedation ^m^ | 0.94 (0.64-1.37) | 0.74 | 0.70 (0.44-1.10) | 0.12 |
| MEQ, per 10 units ^n^ | 1.18 (1.13-1.24) | <0.01 | 1.19 (1.13-1.24) | <0.01 |
| Regional analgesia ^o^ | 1.39 (0.91-2.11) | 0.13 | 1.51 (0.95-2.39) | 0.078 |
| Delirium ^p^ | 1.79 (0.94-3.39) | 0.075 | NC | NC |
| Fever ^q^ | 1.39 (0.93-2.09) | 0.11 | NC | NC |
| Leukocytosis, per 10 units ^r^ | 1.02 (0.76-1.36) | 0.92 | 1.21 (0.89-1.63) | 0.22 |
| CRP, per 10 units ^s^ | 1.00 (0.99-1.02) | 0.58 | 1.00 (0.98-1.02) | 0.92 |

NC not calculated

^a^ Body Mass Index in kg/m^2^.

^b^ No diagnosed diseases prior ICU admission.

^c^ Diabetes mellitus diagnosed prior to ICU admission.

^d^ Malignant tumor or leukemia diagnosed prior to ICU admission.

^e^ Psychiatric disease defined as fulfilling ICD10 diagnostic criteria prior to ICU admission.

^f^ Persistent pain (nociceptive, neuropathic, or unspecified) diagnosis prior to ICU admission.

^g^ Persistent pain described as nociceptive (pain arising from actual or threatened non-neural tissue damage) present prior to ICU admission.

^h^ Persistent pain described as neuropathic (neurological lesion or disease and neuroanatomically plausible pain distribution) diagnosed prior to ICU admission.

^i^ Regular use of prescription opioids prior to ICU admission.

^j^ Admission after surgery, postoperative complications, bleeding, perioperative emergencies, pancreatitis, acute liver failure, trauma.

^k^ Simplified Acute Physiology Score II.

^l^ Noradrenalin administered > 0.1 µg/kg/min over 1 hour per calendar day.

^m^ Continuous administration of a sedative agent (propofol, benzodiazepines, α^2^ -agonists, barbiturates, S-ketamine or inhalation agent) for over 6 hours in calendar day in ICU (excluding sedatives administered outside of ICU).

^n^ Opioid dose in MEQ (oral morphine equivalents) administered in ICU per calendar day.

^o^ Local, regional or neuraxial anesthesia administered in ICU per calendar day.

^p^ Intensive Care Delirium Screening Checklist ≥ 4 points during calendar day in ICU

^q^ Body temperature exceeding 38 degrees Celsius at any time during calendar day.

^r^ Leukocyte count > 8.2^9^/l during calendar day in ICU.

^s^ CRP (C Reactive protein) > 100 mg/l during calendar day ICU.

**Table S9C Results of the mixed effects logistic regression models of pain at rest during the whole period of follow-up in the ICU, excluding elective postoperative patients. Variables were selected for the adjusted model based on clinical relevance and the results of the univariable model.**

|  | **Unadjusted model** | | **Adjusted model** | |
| --- | --- | --- | --- | --- |
| **Variable** | **OR (95% CI)** | **P-value** | **OR (95% CI)** | **P-value** |
| Age | 0.49 (0.29-0.85) | 0.01 | 1.30 (0.70-2.42) | 0.41 |
| Female | 1.30 (0.92-1.85) | 0.14 | 1.54 (1.08-2.21) | 0.017 |
| BMI ^a^ | 1.01 (0.98-1.04) | 0.50 | NC | NC |
| No comorbidities ^b^ | 0.88 (0.63-1.22) | 0.44 | NC | NC |
| Diabetes ^c^ | 1.20 (0.80-1.79) | 0.38 | 1.44 (0.97-2.13) | 0.074 |
| Cancer ^d^ | 1.02 (0.65-1.62) | 0.92 | NC | NC |
| Psychiatric disorder ^e^ | 1.77 (0.93-3.37) | 0.081 | 1.28 (0.66-2.48) | 0.47 |
| Persistent pain ^f^ | 1.16 (0.73-1.83) | 0.53 | NC | NC |
| Nociceptive pain ^g^ | 1.13 (0.67-1.92) | 0.65 | 0.87 (0.51-1.50) | 0.62 |
| Neuropathic pain ^h^ | 1.40 (0.66-2.94) | 0.38 | 1.29 (0.59-2.81) | 0.52 |
| Opioid use ^i^ | 0.76 (0.27-2.10) | 0.59 | 0.84 (0.25-2.80) | 0.78 |
| Surgical diagnosis at admission ^j^ | 2.84 (2.07-3.91) | <0.01 | 2.24 (1.58-3.18) | <0.01 |
| SAPS II, per 10 units ^k^ | 1.00 (0.78-1.29) | 0.98 | NC | NC |
| Vasopressor ^l^ | 1.19 (0.95-1.50) | 0.13 | 0.84 (0.65-1.09) | 0.19 |
| Sedation ^m^ | 1.15 (1.12-1.18) | <0.01 | 1.16 (1.12-1.19) | <0.01 |
| MEQ, per 10 units ^n^ | 1.66 (1.25-2.21) | <0.01 | NC | NC |
| Regional analgesia ^o^ | 1.11 (0.83-1.48) | 0.49 | NC | NC |
| Delirium ^p^ | 1.39 (1.08-1.79) | 0.011 | NC | NC |
| Fever ^q^ | 1.06 (0.89-1.27) | 0.52 | 1.08 (0.90-1.30) | 0.42 |
| Leukocytosis, per 10 units ^r^ | 0.80 (0.71-0.89) | <0.01 | 0.87 (0.77-1.00) | 0.042 |
| CRP, per 10 units ^s^ | 1.01 (1.00-1.02) | 0.26 | 1.00 (0.98-1.01) | 0.42 |

NC not calculated

^a^ Body Mass Index in kg/m^2^.

^b^ No diagnosed diseases prior ICU admission.

^c^ Diabetes mellitus diagnosed prior to ICU admission.

^d^ Malignant tumor or leukemia diagnosed prior to ICU admission.

^e^ Psychiatric disease defined as fulfilling ICD10 diagnostic criteria prior to ICU admission.

^f^ Persistent pain (nociceptive, neuropathic, or unspecified) diagnosis prior to ICU admission.

^g^ Persistent pain described as nociceptive (pain arising from actual or threatened non-neural tissue damage) present prior to ICU admission.

^h^ Persistent pain described as neuropathic (neurological lesion or disease and neuroanatomically plausible pain distribution) diagnosed prior to ICU admission.

^i^ Regular use of prescription opioids prior to ICU admission.

^j^ Admission after surgery, postoperative complications, bleeding, perioperative emergencies, pancreatitis, acute liver failure, trauma.

^k^ Simplified Acute Physiology Score II.

^l^ Noradrenalin administered > 0.1 µg/kg/min over 1 hour per calendar day.

^m^ Continuous administration of a sedative agent (propofol, benzodiazepines, α^2^ -agonists, barbiturates, S-ketamine or inhalation agent) for over 6 hours in calendar day in ICU (excluding sedatives administered outside of ICU).

^n^ Opioid dose in MEQ (oral morphine equivalents) administered in ICU per calendar day.

^o^ Local, regional or neuraxial anesthesia administered in ICU per calendar day.

^p^ Intensive Care Delirium Screening Checklist ≥ 4 points during calendar day in ICU

^q^ Body temperature exceeding 38 degrees Celsius at any time during calendar day.

^r^ Leukocyte count > 8.2^9^/l during calendar day in ICU.

^s^ CRP (C Reactive protein) > 100 mg/l during calendar day ICU.

**Table S10. Missingness of the variables included in the 3-day follow up regression model for the whole cohort when the combined pain outcome is available.**

| **Variable** | **Day 1 n, (%)** | **Day 2 n, (%)** | **Day 3 n, (%)** |
| --- | --- | --- | --- |
| Dexmedetomidine | 0 (0.0%) | 0 (0.0%) | 0 (0.0%) |
| CRP, per 10 units^a*^ | 191 (26.9%) | 12 (1.7%) | 2 (0.3%) |
| Diabetes^b^ | 0 (0.0%) | 0 (0.0%) | 0 (0.0%) |
| Age | 1 (0.1%) | 1 (0.1%) | 1 (0.1%) |
| Opioid use^c^ | 0 (0.0%) | 0 (0.0%) | 0 (0.0%) |
| Neuropathic pain^d^ | 0 (0.0%) | 0 (0.0%) | 0 (0.0%) |
| Nociceptive pain^e^ | 0 (0.0%) | 0 (0.0%) | 0 (0.0%) |
| Leukocytosis, per 10 units^f^* | 140 (19.7%) | 8 (1.1%) | 2 (0.3%) |
| MEQ, per10 units^g^ | 0 (0.0%) | 0 (0.0%) | 0 (0.0%) |
| Female | 0 (0.0%) | 0 (0.0%) | 0 (0.0%) |
| Persistent pain before or at the admission^h^ | 0 (0.0%) | 0 (0.0%) | 0 (0.0%) |
| Psychiatric disorder^i^ | 0 (0.0%) | 0 (0.0%) | 0 (0.0%) |
| Regional analgesia^j^ | 0 (0.0%) | 2 (0.3%) | 1 (0.1%) |
| SAPSII, per10 units^k^ | 9 (1.3%) | 9 (1.3%) | 5 (0.7%) |
| Sedation^l^ | 1 (0.1%) | 1 (0.1%) | 1 (0.1%) |
| Surgical reason for admission^m^ | 0 (0.0%) | 0 (0.0%) | 0 (0.0%) |

^*^ The higher missingness on day one reflects routine clinical practice in elective surgical patients, in whom preoperative laboratory samples are taken prior to surgery and not repeated on the day of the procedure.

^a^ CRP (C Reactive protein) > 100 mg/l during calendar day ICU.

^b^ Diabetes mellitus diagnosed prior to ICU admission.

^c^ Regular use of prescription opioids prior to ICU admission.

^d^ Persistent pain described as nociceptive (pain arising from actual or threatened non-neural tissue damage) present prior to ICU admission.

^e^ Persistent pain described as neuropathic (neurological lesion or disease and neuroanatomically plausible pain distribution) diagnosed prior to ICU admission.

^f^ Leukocyte count > 8.2^9^/l during calendar day in ICU.

^g^ Opioid dose in MEQ (oral morhine equivalents) administered in ICU per calendar day.

^h^ Persistent pain (nociceptive, neuropathic, or unspecified) diagnosis prior to ICU admission.

^i^ Psychiatric disease defined as fulfilling ICD10 diagnostic criteria prior to ICU admission.

^j^ Simplified Acute Physiology Score II.

^k^ Local, regional or neuraxial anesthesia administered in ICU per calendar day.

^l^ Continuous administration of a sedative agent (propofol, benzodiazepines, α^2^ -agonists, barbiturates, S-ketamine or inhalation agent) for over 6 hours in calendar day in ICU (excluding sedatives administered outside of ICU).

^m^ Admission after surgery, postoperative complications, bleeding, perioperative emergencies, pancreatitis, acute liver failure, trauma.

**Figure S1 Visualization of the dichotomized pain at rest variable during the follow up period in the ICU for the subgroup of surgical patients. A) Trajectories of pain shown as patient counts. B) Daily distribution of pain in surgical patient subgroup treated in ICU per evaluation day.**

**Figure S2 Visualization of the dichotomized pain at rest variable during the follow up period in the ICU for the subgroup of non-surgical patients. A) Trajectories of pain shown as patient counts. B) Daily distribution of pain in non-surgical patient subgroup treated in ICU per evaluation day.**

**Figure S3 Visualization of the dichotomized pain at rest variable during the follow up period in the ICU for the subgroup of patients aged 64 years or older. A) Trajectories of pain shown as patient counts. B) Daily distribution of pain in subgroup of patients ≥ 64 years old treated in ICU per evaluation day.**

**Figure S4 Visualization of the dichotomized pain at rest variable during the follow-up period in the ICU for subgroup of patients aged under 64 years. A) Trajectories of pain shown as patient counts. B) Daily distribution of pain in subgroup of patients aged < 64 years treated in the ICU per evaluation day.**

**Figure S5 Visualization of the dichotomized pain at rest variable during follow-up period in the ICU for subgroup of male patients. A) Trajectories of pain shown as patient counts. B) Daily distribution of pain in subgroup of male patients treated in the ICU per evaluation day.**

**Figure S6. Visualization of the dichotomized pain at rest variable during follow-up period in the ICU for subgroup of female patients. A) Trajectories of pain shown as patient counts. B) Daily distribution of pain in subgroup of female patients treated in the ICU per evaluation day.**

**Figure S7. Visualization of the dichotomized pain at rest variable during follow-up period in the ICU for subgroup of opioid-naive patients (no prior opioid use before ICU admission). A) Trajectories of pain shown as patient counts. B) Daily distribution of pain in subgroup of opioid-naive patients treated in the ICU per evaluation day.**

**Figure S8. Visualization of the dichotomized pain at rest variable during follow-up period in the ICU for subgroup of patients regularly using opioids before ICU admission. A) Trajectories of pain shown as patient counts. B) Daily distribution of pain in subgroup of patients regularly using opioids before ICU admission treated in the ICU per evaluation day.**
